# Intraspecies Competition for Niches in the Distal Gut Dictate Transmission during Persistent *Salmonella* Infection

**DOI:** 10.1371/journal.ppat.1004527

**Published:** 2014-12-04

**Authors:** Lilian H. Lam, Denise M. Monack

**Affiliations:** Department of Microbiology and Immunology, Stanford University School of Medicine, Stanford, California, United States of America; University of Michigan Medical School, United States of America

## Abstract

In order to be transmitted, a pathogen must first successfully colonize and multiply within a host. Ecological principles can be applied to study host-pathogen interactions to predict transmission dynamics. Little is known about the population biology of *Salmonella* during persistent infection. To define *Salmonella enterica* serovar Typhimurium population structure in this context, 129SvJ mice were oral gavaged with a mixture of eight wild-type isogenic tagged *Salmonella* (WITS) strains. Distinct subpopulations arose within intestinal and systemic tissues after 35 days, and clonal expansion of the cecal and colonic subpopulation was responsible for increases in *Salmonella* fecal shedding. A co-infection system utilizing differentially marked isogenic strains was developed in which each mouse received one strain orally and the other systemically by intraperitoneal (IP) injection. Co-infections demonstrated that the intestinal subpopulation exerted intraspecies priority effects by excluding systemic *S.* Typhimurium from colonizing an extracellular niche within the cecum and colon. Importantly, the systemic strain was excluded from these distal gut sites and was not transmitted to naïve hosts. In addition, *S.* Typhimurium required hydrogenase, an enzyme that mediates acquisition of hydrogen from the gut microbiota, during the first week of infection to exert priority effects in the gut. Thus, early inhibitory priority effects are facilitated by the acquisition of nutrients, which allow *S.* Typhimurium to successfully compete for a nutritional niche in the distal gut. We also show that intraspecies colonization resistance is maintained by *Salmonella* Pathogenicity Islands SPI1 and SPI2 during persistent distal gut infection. Thus, important virulence effectors not only modulate interactions with host cells, but are crucial for *Salmonella* colonization of an extracellular intestinal niche and thereby also shape intraspecies dynamics. We conclude that priority effects and intraspecies competition for colonization niches in the distal gut control *Salmonella* population assembly and transmission.

## Author Summary

*Salmonella enterica* serovars infect various mammalian hosts, causing disease ranging from self-limiting diarrhea to persistent systemic infections such as typhoid fever. Here we investigated the impact of an established intestinal *S.* Typhimurium population on fecal shedding in the presence of another challenging strain. This scenario arises during host-to-host transmission, as well as during chronic host-adapted infections when systemic *Salmonella* reseed the intestinal tract to be transmitted in feces. In a mouse model of persistent *Salmonella* infection, we found that distinct subpopulations formed in intestinal and systemic tissues. Expansion of the intestinal subpopulation was responsible for increases in fecal shedding, rather than increased secretion of systemic *Salmonella*. Furthermore, the *Salmonella* that initially colonized the gut excluded challengers from the cecum, colon, and feces. A challenging systemic strain could only be shed upon ablation of the established intestinal strain. This intraspecies colonization resistance requires *Salmonella* hydrogenase-mediated invasion of the distal gut and is maintained by the virulence effectors SPI1 and SPI2. We describe novel observations indicating that *Salmonella* virulence effectors that have been shown to subvert the host immune response and microbiota, also play a role in intraspecies competition for colonization of transmission niches.

## Introduction

The *Salmonella enterica* serovars are important pathogens that cause disease ranging from a self-limiting gastroenteritis to persistent systemic infections. The human-adapted *Salmonella enterica* Typhi and Paratyphi serovars are the causative agents of typhoid fever, and penetrate the intestinal epithelium to disseminate to systemic tissues [1]. Approximately 1–6% of infected patients become chronic carriers and serve as the reservoir of disease, remaining asymptomatic while excreting *Salmonella* in their stool [1], [2]. *S.* Typhimurium causes a typhoid-like disease in mice, but also infects a wide-range of mammalian hosts, including livestock [3], [4]. *S.* Typhimurium is a major cause of foodborne diarrheal disease in humans, but can also cause invasive non-typhoidal *Salmonella* (NTS) disease in immunocompromised individuals [5], [6]. NTS can persist in the gastrointestinal tract and be excreted in feces in certain patients [7], with elevated levels of NTS fecal shedding associated with antibiotic therapy [8]. Surprisingly little is known about *Salmonella* fecal shedding dynamics, particularly during persistent infection. However, this aspect of the *Salmonella* life cycle is fundamentally important for understanding transmission to new hosts.

Transmission of this enteric pathogen occurs via the fecal-oral route. During invasive disease with host-adapted serovars, *Salmonella* invade the Peyer's patches (PP) in the small intestine and breach the epithelium. Trafficking through the blood and lymphatics results in systemic dissemination of the pathogen to the mesenteric lymph nodes (mLN), bone marrow, spleen, liver, and gallbladder [9]. It is thought that systemic *Salmonella* in gallbladder bile secretions reseed the small intestine to be transmitted in feces [1], [10]. However, the fate of the initial invading *Salmonella* in the intestine and whether they contribute to fecal shedding has not been determined. A deeper understanding of the within-host population biology of *Salmonella* infections is crucial for determining treatment strategies and preventing spread. The mammalian host can be viewed as an ecosystem, with different tissues functioning as interconnected habitats. In this landscape, pathogens develop into population structures based on processes of dispersal, diversification, environmental selection, and coevolution within the host [11]. During host-to-host spread, each individual acts as an independent ecosystem, and a pathogen must adapt to a new environment in order to be successfully transmitted. Principles in ecology can thus be applied to explain and predict the resulting infection dynamics [11], [12].

Since host-adapted *Salmonella* serovars first enter the gastrointestinal tract before spreading to systemic tissues, we hypothesized that distinct groups of communities would assemble within these two host compartments. In population ecology, this is referred to as a subpopulation, or a local group of individuals that interact within a certain habitat [13]–[17]. A metapopulation then consists of a collection of subpopulations with various interactions and rates of dispersal between their habitats. Indeed, studies utilizing tagged isogenic strains have revealed formation of metapopulations in other systemic infections. Due to differing replication rates and dispersal routes within host tissues, independent pathogen subpopulations form during *Listeria monocytogenes*, *Yersinia pseudotuberculosis*, and uropathogenic *Escherichia coli* infections [18]–[21], although the impact of these subpopulations on transmission is unknown.

Wild-type isogenic tagged *Salmonella* (WITS) strains have been developed to resolve the early kinetics of acute infection in the susceptible C57BL/6 mouse background. In the streptomycin-treated diarrhea model, WITS were applied to generate a mathematical model describing replication and immune clearance of *Salmonella* in the cecal lymph node 24 hours post-infection [22]. Analysis of an intravenous model of infection revealed that concomitant death and rapid bacterial replication resulted in the formation of independent WITS subpopulations in the liver and spleen, although hematogenous mixing led to the homogenization of these systemic communities after 48 hours [23]. A study of early dissemination determined that founder bacteria initiated infection independently in Peyer's patches and systemic compartments 4 days post-infection [24]. However, the WITS technique has not been utilized to dissect the spatiotemporal population dynamics during chronic infections. It is not known whether different subpopulations of *Salmonella* form during persistent infection, or how they contribute to the pool of *Salmonella* that is ultimately shed in the feces. Furthermore, it is important to determine whether *Salmonella* that are carried long-term in systemic tissues and/or in the gallbladder contribute to fecal shedding in the presence of a previously established intestinal subpopulation. The effect of an established intestinal subpopulation on subsequent super-infections is also unclear. However, this scenario could arise in endemic regions and outbreaks, and therefore has implications on human disease and livestock husbandry. It is also unclear whether humans can be co-infected with multiple *Salmonella* strains due to difficulties in obtaining consistent patient samples, but this scenario could arise in endemic regions and outbreaks.

Studies in ecology have determined that immigration order dictates community structure through a priority effect, in which early colonization affords one member an advantage over future colonizers [25]–[27]. These competitive interactions are often mediated by resource availability [26]–[28]. Darwin's naturalization hypothesis posits that challenging species are more successful in habitats in which their close relatives are absent [29], as the more closely related they are, the more strongly they will compete for the same resources. Following this logic, we hypothesized that different subpopulations of *Salmonella* will compete for colonization of niches important for fecal shedding.

In this study, we employed tagged isogenic *S.* Typhimurium strains in a mouse model of persistent systemic infection. We show that a *Salmonella* metapopulation structure forms during persistent infection, with distinct subpopulations in intestinal and systemic tissues. We further found that established subpopulations of intestinal *Salmonella* colonize crucial extracellular niches in the cecum and colon that are required for fecal shedding. Systemic *Salmonella* from the gallbladder, as well as challenging strains from other infected donor mice, are excluded from the distal gut niche in a novel observation of intraspecies colonization resistance by an enteropathogen. *Salmonella* hydrogenase, an enzyme that mediates acquisition of microbiota-derived hydrogen [30], is required to exert priority effects in this crucial transmission niche. In addition, we demonstrate that maintenance of this intraspecies colonization resistance is dependent on the *Salmonella* pathogenicity islands SPI-1 and SPI-2 during persistent infection.

## Results

### A tagged strain approach reveals formation of *Salmonella* subpopulations during persistent infection

**Figure 1:**
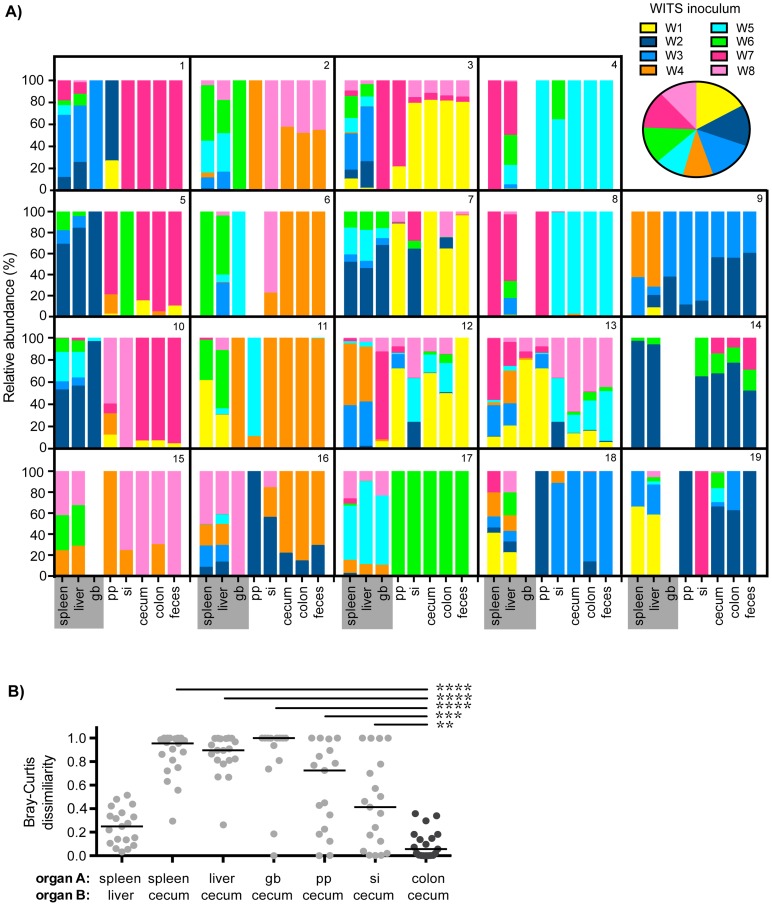
Distinct *Salmonella* subpopulations arise in systemic and intestinal tissues during chronic infection. Mice were oral gavaged with 10^8^ *S.* Typhimurium consisting of an equal mixture of 8 WITS strains. WITS profiles in various tissues were determined by qPCR. Data shown represent 3 independent experiments (n = 19). A) Spleen, liver, gallbladder (gb), PP (pp), small intestine (si), cecum, colon, and fecal samples were collected after 35 days of infection. Bars depict the relative abundance of each WITS strain in different tissue compartments, each box contains the WITS profile for an individual mouse (1–19). Systemic tissues are highlighted in gray. *Inset:* composition of the WITS inoculum. B) Bray-Curtis dissimilarity values of WITS relative abundances in organ A versus organ B. Each circle represents an individual mouse (n = 19), lines represent medians. The lowest median dissimilarity in the depicted comparisons was observed between the colon and cecum (black circles). Intergroup differences were evaluated by paired t-tests. ** p = 0.0024, *** p = 0.0002, **** p<0.0001.

To define the *Salmonella* population structure that arises during chronic infection, we employed a previously established tagged strain approach using a mixture of barcoded, phenotypically equivalent *S.* Typhimurium strains [23]. These *Salmonella* wild-type isogenic tagged strains (WITS) each carry a unique 40 base pair tag in between the *malX* and *malY* pseudogenes, are equally fit, and have been applied to studies of acute infection [23]. Utilizing these previously published sequence tags, we constructed 8 WITS strains in the *S.* Typhimurium SL1344 background (W1–W8; Table S1) and confirmed each strain to be equally fit when grown in broth culture (Figure S1A). 129X1/SvJ mice, which possess a wild-type *Nramp1* allele and can be persistently colonized with *S.* Typhimurium [31]–[33], were orally inoculated with 10^8^ colony forming units (CFU) of an equal mixture of strains W1–W8 (Figure S1B–C). Total WITS CFU were enumerated by plating (Figure S1D) and qPCR was performed to determine the WITS abundances in systemic (spleen, liver, gallbladder) and intestinal (PP, small intestine, cecum, colon, feces) sites after 35 days of infection. Individual mice had WITS profiles that were distinct from other animals, with certain WITS comprising the majority of *Salmonella* found within infected tissues that varied on a mouse-by-mouse basis (Figure 1A). However, combined analysis of all infected mice revealed that all 8 WITS strains were represented in every tissue compartment (Figure 1A) and there was no statistically significant difference between the relative abundances of the WITS strains in each of the tissues, indicating all 8 WITS are equally represented *in vivo* (Table S2, one-way ANOVA and Kruskal-Wallis tests). A control experiment in which 4 of the 8 WITS were underrepresented in the inoculum resulted in their subsequent underrepresentation within infected tissues (Figure S2), indicating that these 4 WITS did not have any fitness advantage during infection.

The WITS compositions in systemic and intestinal tissues were compared in order to determine whether *Salmonella* subpopulations arose after 35 days of persistent infection, a time after which the bacteria have breached the intestinal epithelium and have spread systemically to the liver and spleen. The strain composition within individual mice varied depending on the site of infection (Figure 1A). In order to quantify potential differences in WITS abundances, we utilized a Bray-Curtis dissimilarity statistic, which has been commonly used in community abundance analyses in ecology and studies of the microbiota [34]–[36]. This calculation was applied to our model to obtain population-level distance values of WITS compositions in different sites. Bray-Curtis values were calculated between the WITS relative abundances of two tissues (see Materials and Methods), in which a score of 0 indicates an identical WITS profile in both organs and a score of 1 indicates completely dissimilar populations. A dissimilarity matrix was calculated for all tissue comparisons (Table 1). The subpopulation in the liver closely matched that of the spleen with a low mean dissimilarity score of 0.248 (Figure 1B, Table 1), which is consistent with these environments being highly connected by migration pathways through the bloodstream and/or lymphatics.

**Table 1:** Bray-Curtis dissimilarity values of WITS relative abundances within murine tissues during persistent infection.

	SYSTEMIC			PROXIMAL GUT		DISTAL GUT		
	spleen	liver	gb	pp	si	cecum	colon	feces
spleen	0	*0.248*	0.602	0.848	0.801	0.858	0.816	0.873
liver	*0.248*	0	0.619	0.852	0.798	0.857	0.817	0.881
gb	0.602	0.619	0	0.775	0.772	0.821	0.811	0.842
pp	0.848	0.852	0.775	0	0.728	0.574	0.575	0.562
si	0.801	0.798	0.772	0.728	0	0.442	0.416	0.446
cecum	0.858	0.857	0.821	0.574	0.442	0	***0.101***	***0.077***
colon	0.816	0.817	0.811	0.575	0.416	***0.101***	0	***0.129***
feces	0.873	0.881	0.842	0.562	0.446	***0.077***	***0.129***	0

Mice infected with an equal mixture of 8 WITS strains as described in Figure 1. WITS relative abundances were determined by qPCR, dissimilarity scores between relative abundances in two sites, organ A versus organ B, were calculated by Bray-Curtis analyses (see Materials and Methods). Rows = organ A, columns = organ B. Organ B comparisons were organized into columns based on classification as systemic tissues (spleen, liver, gb), proximal gut sites (pp, si), or distal gut samples (cecum, colon, feces). Scores were computed for each individual mouse (data representative of 3 independent experiments, n = 19), table reflects the mean dissimilarity value for all animals. A Bray-Curtis value of 0 indicates identical WITS profiles (*e.g.* spleen versus spleen), while a score of 1 represents completely dissimilar WITS abundances. *Italicized:* Lowest dissimilarity score between systemic tissues (spleen versus liver). *Bold and italicized:* The three lowest dissimilarity scores observed in distal gut samples.

In addition to colonizing systemic sites, *Salmonella* persisted within intestinal tissues for 35 days. However, in contrast to the spleen and liver, which contained an average of 3–4 WITS, the intestinal tissues were colonized by 1–2 strains (Figure 1A). This suggested that while there was some bottlenecking in dissemination to systemic sites, stronger selection pressures likely existed within intestinal tissues. Further analysis of the WITS profiles indicated that the strain compositions in proximal gut tissues (PP and small intestine) were dissimilar from those present in distal gut tissues (cecum and colon, Figure 1A), with dissimilarity scores of 0.416–0.575 (Figure 1B, Table 1, Figure S3A). In contrast, the WITS composition in the cecum and colon were very similar with a score of 0.101 (Figure 1B, Table 1), which was significantly lower than the dissimilarity scores observed in the proximal gut (Figure S3A). Together, these data suggest that during persistent infection, different subpopulations of *Salmonella* form between proximal and distal gut tissues.

It is thought that *Salmonella* in the liver and gallbladder reseed the intestinal tract via bile, followed by subsequent shedding in the feces. If the bile ducts provided highly connected migration pathways between these sites, the WITS profiles should be similar between systemic and intestinal tissues. Although not all of the mice were colonized by *Salmonella* in the gallbladder (Figure 1A), the WITS profiles in the gallbladder were most similar to the compositions of the spleen and liver from these mice (Table 1, Figure S3B). In contrast, the WITS compositions in the gallbladder were very different from the composition within the intestinal tissues (Figure 1B, Table 1, Figure S3B). In addition, the WITS compositions in the distal gut were distinct from those in the systemic tissues with high dissimilarity scores >0.816 (Figure 1B, Table 1). Collectively, analysis of the WITS compositions in various compartments within each infected mouse demonstrate that spatially delimited *Salmonella* subpopulations form during persistent infection, with systemic organs containing populations that are distinct from those in intestinal tissues.

### Increases in fecal shedding are attributed to clonal expansion of colonic *Salmonella*

**Figure 2:**
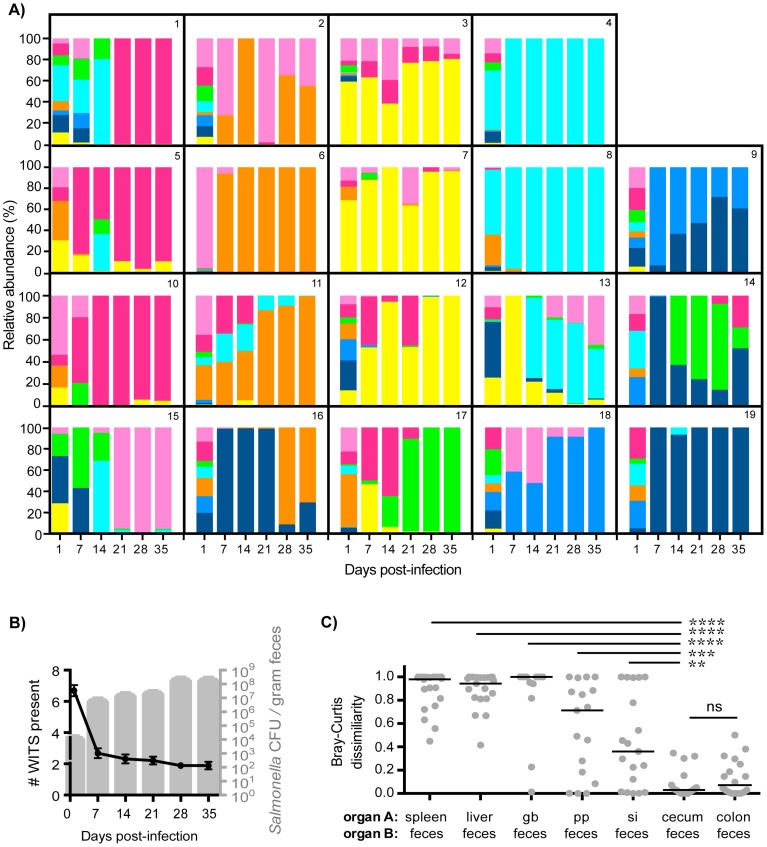
Clonal expansion of cecal and colonic *Salmonella* is responsible for increased fecal shedding. Fecal shedding of *S.* Typhimurium in mice orally infected with an equal mixture of 8 WITS strains over 35 days of infection. Data shown is representative of 3 independent experiments (n = 19). A) Profiles of WITS shed in feces at the indicated time points post-infection as determined by qPCR. Individual mice were tracked, and the identifying mouse number correlates to the same animal in Figure 1. B) *Left y-axis:* number of WITS strains present at the specified time points post-infection (black line: mean, SD). *Right y-axis:* total *Salmonella* CFU enumerated from fresh fecal pellets collected throughout infection (gray columns: mean, SD). C) Bray-Curtis dissimilarity values between WITS relative abundances in organ A and organ B (feces). Each circle represents an individual mouse (n = 19), lines represent medians. Intergroup differences were evaluated by paired t-tests. ns = not significant, ** p = 0.0041, *** p = 0.0010, **** p<0.0001.

Since host-to-host transmission requires high levels of *Salmonella* shed in the feces [4], [33], we wished to elucidate the kinetics and population dynamics of *Salmonella* shedding. Fecal samples were collected at various time points throughout the 35-day infection period (Figure 2A). An average of 6–7 WITS were present in feces after one day of infection, indicating some initial bottlenecking effects in the oral infection route may have occurred (Figure 2A–B). However, even greater dynamic changes in WITS compositions were observed at early time points in infection, with different strains shed at 7 and 14 days post-infection compared to day 1 (Figure 2A). Importantly, there was a dramatic decrease in the number of strains detected in the feces to an average of 1–2 WITS, which did not change during the 35-day infection (Figure 2A–B). Importantly, the sharp decrease in the number of strains shed in the feces on day 7 correlated with an increase in total fecal *Salmonella* CFU (Figure 2B), suggesting that clonal expansion of dominant WITS strains was responsible for increased fecal shedding.

To ascertain the tissue compartment that served as the source of clonal *Salmonella* expansion, we compared the WITS relative abundance profiles of the feces to both systemic and intestinal tissues to identify similarities. Although *Salmonella* initially invade the PP, the WITS compositions in the PP compared to the feces were significantly different at 35 days post-infection (Figures 2C, Table 1). In addition, the compositions of the *Salmonella* populations within systemic sites compared to the population composition in the feces were even more dissimilar (Table 1). This further corroborated our earlier finding that distinct *Salmonella* subpopulations arose between systemic and intestinal compartments. Furthermore, we did not observe an increase in the number of WITS strains present during increased fecal shedding (Figure 2B), which would be expected to occur if increased reseeding of systemic *Salmonella* was the source. Instead, these analyses revealed that the WITS profiles in both the cecum and colon very closely matched the composition of *Salmonella* shed in the feces (Figure 2B; Table 1). Importantly, the dissimilarity values between the distal gut sites and the feces were significantly lower than that of any other tissue compartment analyzed (Figure 2C, Table 1, Figure S3). Taken together, our results indicate that a clonal expansion of cecal and colonic *Salmonella* is responsible for the increases in fecal shedding.

### Systemic *Salmonella* are excluded from the distal gut and subsequent fecal shedding

The results of our WITS experiment demonstrated that distinct subpopulations formed in systemic and intestinal tissues by 35 days post-infection (Figure 1). However, even though high Bray-Curtis scores were computed between systemic and distal gut tissues, values were <1 indicating there were small percentages of shared WITS in these sites. One limitation of our mixed inoculum approach was that we could not discern the directionality of dissemination. For example, it could not be determined whether WITS present in the distal gut were part of the initial population or if they arrived secondarily by seeding the intestinal tract from systemic sites. In order to determine the relative contribution of systemic and intestinal strains to fecal shedding, it required a strategy to mark *Salmonella* in these different sites within the host.

To address this, we developed a co-infection model that employed isogenic marked strains rapidly identifiable by differential plating on antibiotics. We used the parental streptomycin-resistant SL1344 strain that has a missense mutation in *hisG*, which is not required for virulence, and an isogenic SL1344-*kan^R^* strain containing a kanamycin resistance cassette inserted at this site (*hisG::aphT*). These strains are equally fit in single and in mixed infections in mice inoculated by oral or IP routes [33]. In our co-infections, each mouse received 10^8^ of one strain by oral inoculation and 10^3^ of the isogenic strain by intraperitoneal (IP) injection. The IP route bypasses the gastrointestinal tract, such that *Salmonella* colonize systemic tissues first [32]. To confirm that successful reseeding occurs in our model, *Salmonella* shedding and tissue burdens were compared in control mice that received single IP infections or those that received single oral infections. Systemic IP-delivered *Salmonella* reseeded the small intestine, where they reached the same range of fecal shedding levels by 14 days post-infection as mice infected orally (Figure S4). However, the oral inoculation route resulted in >1,000-fold more *Salmonella* fecal CFUs 1 day post-infection compared to the IP route, and reached peak fecal shedding levels more rapidly (Figure S4A). Thus, in the co-infection model, the oral strain establishes an infection in the gut before the systemic strain reaches the intestine, allowing us to test the strength of priority effects in *Salmonella* population assembly.

**Figure 3:**
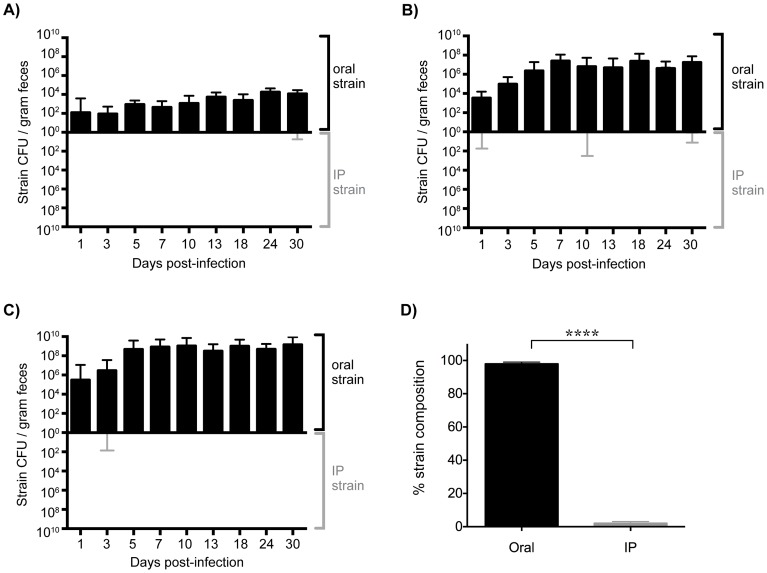
Established gastrointestinal populations of *Salmonella* are exclusively shed in feces. Differentially marked, isogenic *Salmonella* strains were used in co-infections. Mice were given 10^8^ SL1344 by drinking and injected intraperitoneally (IP) with 10^3^ SL1344-*kan^R^* immediately afterwards. The reciprocal combination of strains was also tested and included. A–C) *Salmonella* CFU/gram feces were determined from fecal pellets collected over 30 days of infection. SL1344-*kan^R^* was identified by differential plating or patching onto LB agar containing 40 µg/ml kanamycin. Oral strain CFU (black) are plotted on the top half of the graph and the IP strain CFU (gray) on the bottom. Limit of detection for a single fecal sample is 10 CFU/gram feces. Each plot represents *Salmonella* fecal CFU (median, range) for mice shedding at A) low (n = 8), B) moderate (n = 34), and C) super shedder (n = 12) levels. D) Oral (black) and IP (gray) strain composition of *Salmonella* shed in feces after 30 days of co-infection (mean, SD). Data represent four independent experiments (n = 54). **** p<0.0001, Wilcoxon matched-pairs signed rank test.

Mice injected IP with a single *Salmonella* strain shed this strain in the feces as soon as 1 day post-infection (Figure S4A). This was in contrast to what occurred in mice that had been co-infected orally with an isogenic WT strain (Figure 3). The systemic strain was detected in the feces of only 5 of the 54 mice throughout the 30 days of infection (Figure 3A–C). Importantly, shedding of the systemic strain only occurred on a single day and did not persist. Since mice shed variable levels of *Salmonella* [33], [37], we wondered whether this would influence the ability of the IP strain to be shed. Surprisingly, the oral strain was exclusively shed in the feces of low (<10^4^ CFU/gram), moderate (<10^8^ CFU/gram), and super (≥10^8^ CFU/gram) shedder mice (Figure 3A–C). In addition, when the reciprocal combination of strains (oral: SL1344-*kan^R^*, IP: SL1344) was used the same result was obtained throughout 60 days of infection (Figure 3, Figure S5A). Taken together, these results indicate that the established intestinal strain prevents colonization of the cecum and colon by *Salmonella* disseminating from systemic tissues.

**Figure 4:**
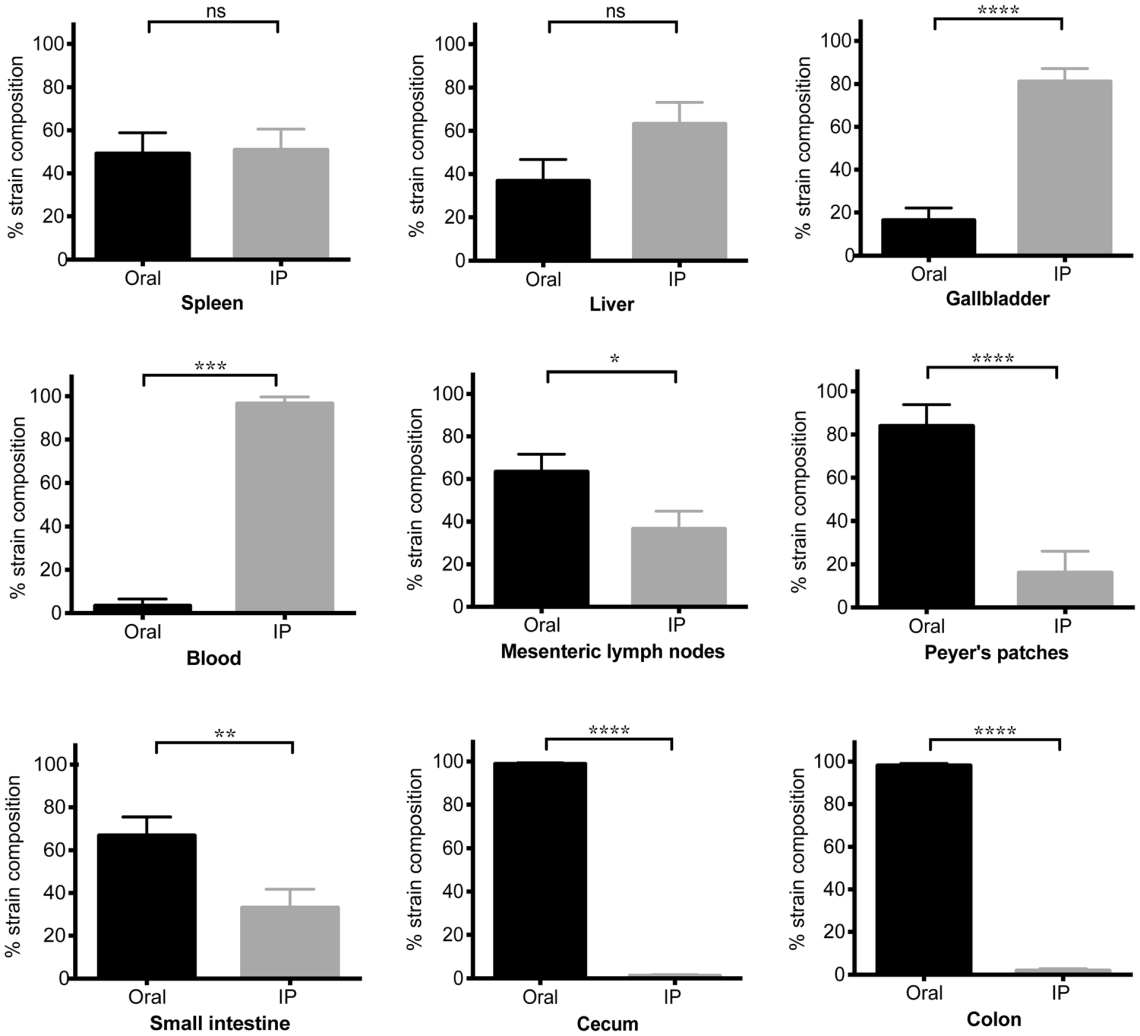
Systemic *Salmonella* are excluded from the distal gut during competition with an established gut strain. Mice were co-infected with SL1344 orally and SL1344-*kan^R^* IP. Animals were sacrificed after 30 days of infection. Specified organs were collected and plated for determination of oral (black) and IP (gray) strain abundance. The reciprocal combination of strains was also tested and included. Data represent three independent experiments (n = 28). Percent strain composition of *Salmonella* in tissues. (mean, SD). ns = non-significant, *p = 0.0159, ** p = 0.0087, ***p = 0.0002, **** p<0.0001, Wilcoxon matched-pairs signed rank tests.

We next wondered what the composition of the *Salmonella* strains were within systemic tissues of mice that had been co-infected for 30 days. In contrast to the cecum and colon, the IP and oral strains were both present within systemic tissues after 30 days of co-infection. The spleen and liver were comprised of similar abundances of both strains (Figure 4), indicating that intestinal *Salmonella* effectively disseminated to systemic sites. Although the orally inoculated strain was present in the gallbladder, the IP strain comprised >80.44% of the total *Salmonella* population in this organ (Figure 4). In addition, the IP strain was present as a minority of the population present in the PP (19%), small intestine (30%), and mLN (38%) (Figure 4). The IP strain was not detected in the cecum and colon in 25 out of 28 mice, and comprised <8% in the remaining animals (Figure 4). Strikingly, the oral strain remained dominant in the cecum and feces during the 60-day infection (Figure S5). Thus, our results from the co-infection model and the WITS analyses suggest that *Salmonella* that are established in the cecum and colon prevent systemic subpopulations from colonizing important niches that are required for fecal shedding.

### Increased *Salmonella* levels in the gallbladder does not lead to fecal shedding of systemic bacteria

One possible explanation for the dominance of the oral strain in the distal gut and feces could be that there is insufficient reseeding of systemic *Salmonella* into the gastrointestinal tract. To test this possibility, we utilized an established gallstone model of infection, in which *S.* Typhimurium biofilm formation on gallstones increased reseeding and subsequent fecal shedding by 1,000-fold [38]. We fed mice a lithogenic diet for 10 weeks to induce gallstone formation that resulted in 1–9 stones/mouse as confirmed by ultrasound imaging (Figure S6). In contrast, mice on a standard diet never developed gallstones (Figure S6). As previously demonstrated, mice with gallstones that were infected with 10^3^ *S.* Typhimurium by IP injection shed >1,000-fold higher levels of bacteria 7 days post-infection compared to control mice (Figure S7).

**Figure 5:**
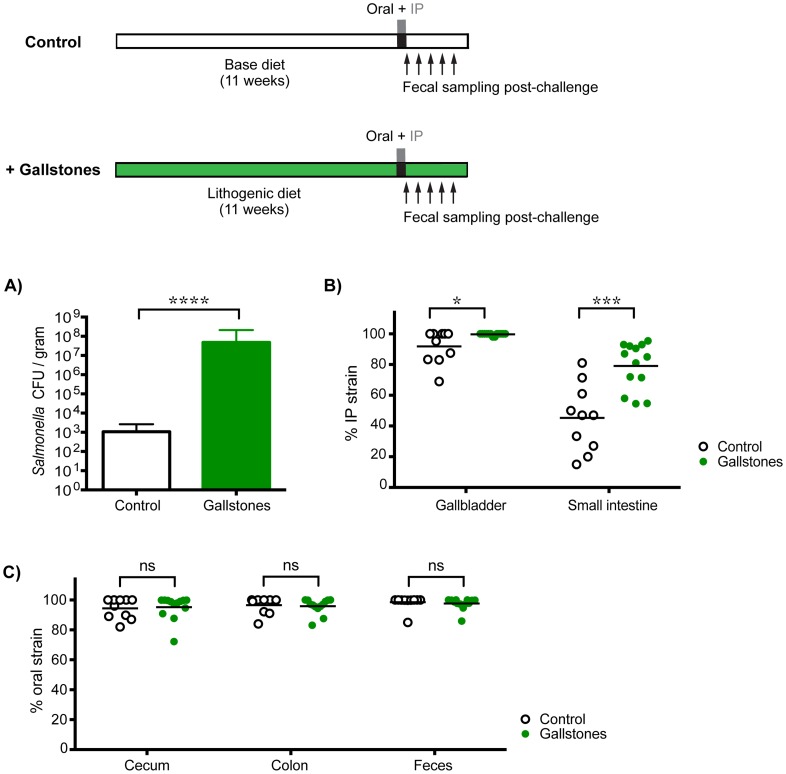
Gallstone-induced increases in systemic reseeding *Salmonella* are insufficient to displace the established population in the cecum and colon. Mice on an 11-week lithogenic diet developed cholesterol gallstones (green, n = 13), while mice on the normal base diet were devoid of gallstones (white, n = 10). All mice were co-infected with 10^8^ SL1344 orally and 10^3^ SL1344-*kan^R^* by IP injection. Feces and specified tissues were collected 14 days post-infection to calculate *Salmonella* burden and strain abundance. A) Total *Salmonella* CFU per gram gallbladder tissue (mean, SD), B) percent abundance of the IP strain in the gallbladder and small intestine, and C) percent abundance of the oral strain in the cecum, colon, and feces were determined in control mice and mice with gallstones. Each circle represents an individual mouse, lines represent means. Data are representative of two independent experiments. ns = non-significant, *p = 0.0224, ***p = 0.0004, ****p<0.0001, unpaired Mann-Whitney tests.

To determine whether increased levels of *S.* Typhimurium in the gallbladder would allow systemic bacteria to colonize the cecum and/or colon, mice with diet-induced gallstones were co-infected orally with SL1344 and IP with SL1344-*kan^R^*. By 14 days post-infection, mice with gallstones had a mean *Salmonella* gallbladder burden >10,000-fold higher than mice without gallstones (Figure 5A). This represented an increase in systemic *Salmonella*, as the gallbladders were exclusively colonized by the IP strain (Figure 5B). In addition, mice with diet-induced gallstones had significantly higher levels of *S.* Typhimurium in the small intestine, indicating that increased numbers of systemic bacteria had reseeded this site (Figure 5B). Despite this drastic increase in the levels of systemic *Salmonella* reseeding the intestine, the established intestinal strain remained dominant in the cecum, colon, and feces (Figure 5C). Taken together, our data suggest that in the presence of an established *Salmonella* strain, systemic *Salmonella* are excluded from colonizing crucial transmission niches in the distal gut.

### The established intestinal strain is resistant to super-colonization regardless of infection route

**Figure 6:**
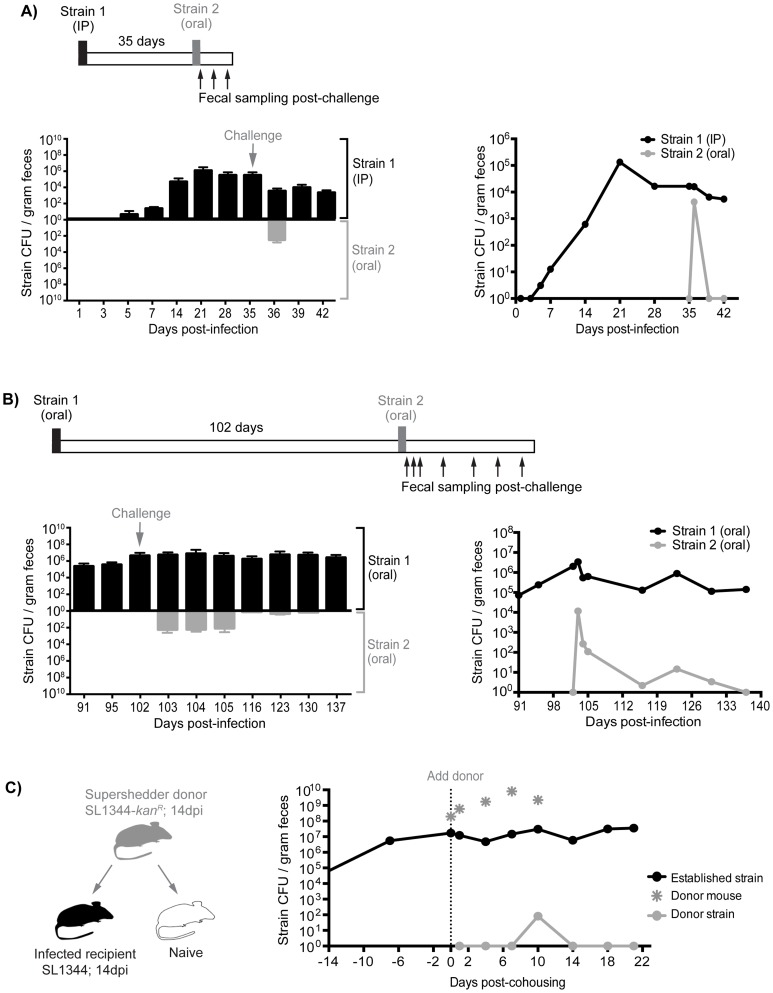
Established strains are resistant to super-colonization regardless of infection route. Sequential infections in mice were performed using SL1344 as the initial strain (Strain 1, black) and SL1344-*kan^R^* as the competing strain (Strain 2, gray). Individual mice were monitored for fecal *Salmonella* shedding at the indicated time points. Pellets were plated to determine total *Salmonella* CFU and to discern strain abundances. Limit of detection for a single fecal sample is 10 CFU. A) Mice were first infected with 10^3^ SL1344 by IP injection. After 35 days, mice were challenged with 10^8^ SL1344-*kan^R^* by drinking. *Left:* Fecal CFU (mean, SD) of established IP and competing (oral) strains. *Right:* Geometric mean of strain CFU in feces. Data are representative of two separate experiments (n = 10). B) Mice were first inoculated with 10^8^ SL1344 (black) by drinking, which established a persistent infection for 102 days. Animals were subsequently challenged with 10^8^ SL1344-*kan^R^* (gray) by drinking. *Left:* Fecal CFU (mean, SD) of established strain SL1344 and challenge strain SL1344-*kan^R^* in feces. *Right:* Geometric mean of strain CFU in feces. Data are representative of two separate experiments (n = 45). C) Mice were first infected orally with 10^8^ of either SL1344 or SL1344-*kan^R^* 14 days prior to cohousing. *Left:* schematic of cohousing experiment; a SL1344-*kan^R^* super shedder donor (gray) was cohoused with SL1344-infected recipient mice (black) and a naïve mouse (white) as a control. *Right:* total *Salmonella* CFU/gram feces in cohoused mice. Gray asterisks indicate shedding levels of the SL1344-*kan^R^* super shedder donor, which was removed after 10 days of co-housing. Lines depict the geometric mean *Salmonella* CFU/gram feces of recipient mice shedding the established SL1344 strain (black) or challenging donor SL1344-*kan^R^* strain. Data are representative of two independent experiments (n = 2 donors, 8 recipients).

Although the presence of gallstones increased the numbers of *S.* Typhimurium in the gallbladder to 10^3^–10^7^ CFU/organ as well as subsequent reseeding of the small intestine, it is possible that these levels were insufficient to compete with the established intestinal subpopulations (Tables S3, S4). Indeed, we have measured the levels of *Salmonella* in gastrointestinal sites and found that there is a range of 10^1^–10^8^ total CFU (Table S3). To address this issue, we performed sequential infections in which resident intestinal *Salmonella* were challenged with a high oral dose of a second strain. First, mice were inoculated with 10^3^ SL1344 by IP injection to establish a systemic infection. This initial *Salmonella* strain was detected in the feces after 5–7 days and was persistently shed for 35 days (Figure 6A). These mice were then super-infected with 10^8^ SL1344-*kan^R^* orally. Although, the orally inoculated strain was detected in the feces 1 day post-infection (dpi), it was not detected in the feces for the remaining 7 days post-oral inoculation (35–42 dpi, Figure 6A). The challenging oral strain was not detected in any systemic or intestinal tissues by 7 days post-challenge (42 dpi, Figure S8A). This demonstrates that super-infecting strains are excluded from colonizing the intestine in the presence of a resident, persistent intestinal *Salmonella* infection, regardless of the route of inoculation. Collectively, our results suggest that there is intraspecies competition for a transmission niche in the distal gut.

### Priority effects govern gut colonization and fecal shedding of *Salmonella*

Based on our evidence of intraspecies competition for a distal gut niche, we proposed that the dominance of established *Salmonella* in the cecum and colon is attributed to priority effects that govern distal gut colonization and subsequent fecal shedding. To test this notion, we performed sequential *S.* Typhimurium infections to evaluate the duration and strength of these competitive interactions. Mice were infected with 10^8^ SL1344 orally, and fecal shedding of *Salmonella* was monitored. All of the mice continued to shed *Salmonella* over the 102 days of infection (Figure 6B). After 102 days, the mice were inoculated orally with 10^8^ CFU of a second competing strain, SL1344-*kan^R^*. The competing strain was detected in the feces during the first 3 days post-infection (Figure 6B). However, by 14 days post-challenge (116 dpi), 28 of the 45 mice were no longer shedding the competing *Salmonella* strain (Figure 6B). Finally, by 35 days post-challenge (137 dpi), the competing strain was not detected in the feces (Figure 6B), intestinal compartments, or systemic tissues of co-infected mice (Figure S8B). The reciprocal strain combinations were also tested: mice were first infected with 10^8^ SL1344-*kan^R^* orally for 60 days before subsequent challenge with 10^8^ SL1344, in which the competing strain was cleared from the feces by 20 days post-challenge (Figure S9A). Thus, this colonization resistance against the same *Salmonella* species was maintained during the chronic stages of infection.

We next sought to determine whether the levels of the initial oral strain (SL1344) in the colon and in the feces would influence the clearance kinetics of the second competing oral strain (SL1344-*kan^R^*). One day after the second oral inoculation, the percentage of the competing strain varied depending on the level of shedding of the resident strain. For example, in mice that were shedding >10^8^ CFU/g feces (super shedder mice), the competing SL1344-*kan^R^* strain comprised 4.88% of the total population on the first day post-secondary inoculation (Figure S9B). In contrast, for low and moderate shedder mice, the competing strain comprised 42.02% and 35.58% of the total population, respectively (Figure S9B). These differences remained significant 5 days after infection with the second competing SL1344-*kan^R^* strain. However, by days 10 and 14, the second SL1344-*kan^R^* strain was no longer detected in the feces of any of the mice (Figure S9B). These results indicate that more robust and rapid priority effects are exhibited in mice that are colonized with higher colonic *Salmonella* loads.

Finally, to determine whether we would see the same intraspecies priority effects in the distal gut during host-to-host transmission, we utilized our previously established model of transmission from an infected, super shedder mouse to uninfected mice in the same cage [33]. In this experiment, the donor mouse was orally infected with SL1344-*kan^R^* and was shedding >10^8^ CFU/g at 14 days post-inoculation (Figure 6C). As a positive control for host-to-host transmission, the donor mouse was co-housed with uninfected mice. Similar to our previous results, naïve mice began shedding SL1344-*kan^R^* within 24 hours and continued to shed even after the donor was removed (Figure S10A). In contrast, recipient mice that had been infected for 14 days with SL1344 required 10 days of cohousing before low levels of the donor strain (<0.02% of all *Salmonella*) were detected in the feces (Figure 6C). In addition, shedding of the donor strain in the previously infected recipient mice was transient, and was not detected in the feces or tissues 10 days post-cohousing (Figure 6C, Figure S8C). Similar results were obtained when a SL1344 super shedder was cohoused with SL1344-*kan* ^R^ infected mice. Cohousing for 12 days was required before the donor strain could be detected in the feces of recipient SL1344-*kan^R^* mice (Figure S10B). The super shedder donor was left in the cage for an additional 6 days before removal, but consistent with previous findings, the donor strain was not detected in the feces of recipient mice by day 23 post-cohousing (Figure S10B). Together, these experiments show that priority effects determine *Salmonella* population assembly in intestinal transmission niches, where established subpopulations exert colonization resistance against incoming challengers.

### Ablation of the established intestinal subpopulation permits distal gut colonization and fecal shedding of systemic *Salmonella*

**Figure 7:**
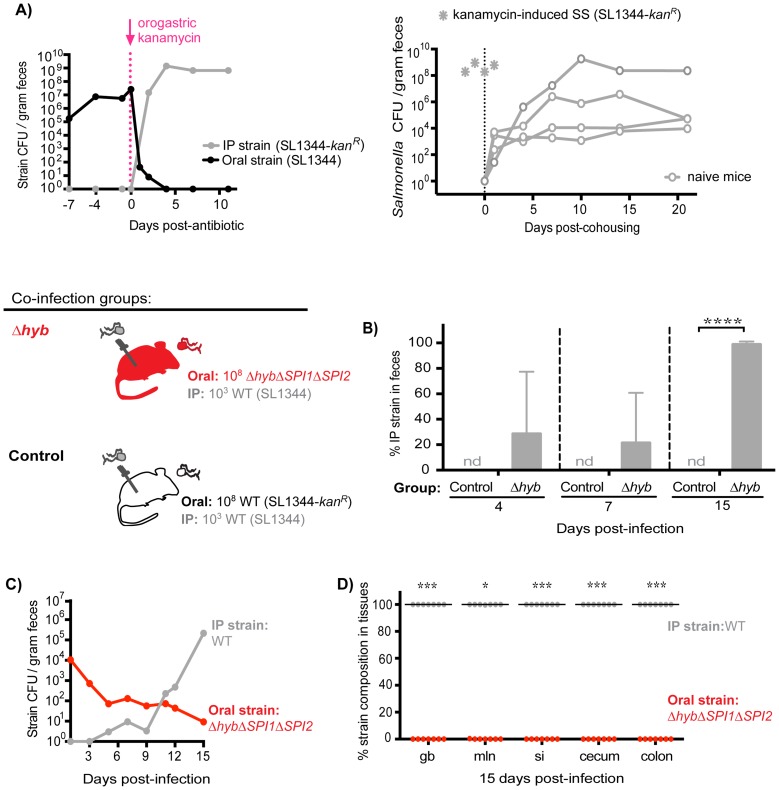
Inhibitory priority effects arise early during infection and are facilitated by *Salmonella* hydrogenase. A) *Left:* Mice were co-infected with 10^8^ SL1344 orally and 10^3^ SL1344-*kan^R^* IP. A single dose of 20 mg kanamycin in 200 µl water was delivered by oral gavage after 7 days of co-infection. Geometric means of oral (black) and IP (gray) strain CFU shed per gram feces. Data are representative of two independent experiments (n = 10). *Right:* A kanamycin-induced supershedder (SS, gray asterisks) shed and transmitted the IP strain exclusively (SL1344-*kan^R^*). Naïve uninfected recipients (open gray circles) began shedding SL1344-*kan^R^* as soon as 1 day post-cohousing, and continued to shed the strain throughout 21 days even after removal of the SS donor. Data are representative of two independent experiments (n = 2 donors, 8 naïve recipients). B) All co-infected mice received 10^3^ WT SL1344 by IP injection (gray *Salmonella*). Mice in the control group received 10^8^ WT SL1344-*kan^R^* orally (white *Salmonella*), and mice in the Δ*hyb* group received 10^8^ *ΔhybΔSPI1*Δ*SPI2* orally (red *Salmonella*). Data are representative of two independent experiments (control n = 10, Δhyb n = 7). Percent of fecal *Salmonella* comprised of the IP strain (WT SL1344) at the indicated days post-infection. Limit of percent IP strain detection was 0.0048% by differential plating, nd = not detected. ****p<0.0001, unpaired Mann-Whitney test. C) *Salmonella* strain composition in feces of Δhyb co-infected mice. Geometric means of *Δhyb*Δ*SPI1*Δ*SPI2* (oral) and WT SL1344 (IP) strains shed in feces over 15 days of co-infection. *D*) Percent of *Salmonella* comprised of *Δhyb*Δ*SPI1*Δ*SPI2* (oral) and WT SL1344 (IP) after 15 days of co-infection. gb = gallbladder, mln = mLN, si = small intestine. Each circle represents an individual animal, lines at medians. Limit of detection was 0.22% by differential plating. *p = 0.0156, ***p = 0.0006, Wilcoxon matched-pairs signed rank tests.

Since the established subpopulation of *Salmonella* in the cecum and colon exerts colonization resistance, we proposed that their removal would allow challengers to occupy vital transmission niches. To test this idea, mice co-infected with 10^8^ SL1344 orally and 10^3^ SL1344-*kan^R^* IP for 7 days were then treated with a single dose of kanamycin. Kanamycin is not absorbed systemically and thus was used to ablate the extracellular, kanamycin-sensitive bacteria in the gastrointestinal tract. Within 24 hours of antibiotic administration, fecal shedding of the established intestinal SL1344 decreased by ∼5 logs (Figure 7A, *left*). Concomitant with the decrease in the established strain, over 10^7^ CFU of the systemic SL1344-*kan^R^* strain was shed per gram of feces (Figure 7A, *left*). By 4 days post-antibiotic treatment, the systemic strain was exclusively shed in the feces (Figure 7A, *left*) and was transmitted to naïve recipients (Figure 7A, *right*). Thus, priority effects arose during the first 7 days of infection, which coincided with the clonal expansion in the distal gut and feces observed in the WITS studies (Figure 2).

Based on these findings, we hypothesized that *Salmonella* strains were competing for limited nutrient or spatial resources within the cecum and colon, which inhibited the ability of systemic strains to colonize the distal gut. We tested this notion by gavaging co-infected mice (SL1344 oral, SL1344-*kan^R^* IP) with 5 mg streptomycin in order to disrupt the microbiota and make more of these resources available [37], [39]–[41]. Both *Salmonella* strains are streptomycin-resistant, and previous studies have shown that streptomycin treatment of infected mice increases *Salmonella* fecal shedding to super shedder levels [33], [37]. We observed that all streptomycin-treated mice became super shedders, yet the increase in fecal *Salmonella* CFU reflected expansion of the oral SL1344 strain (Figure S11). This indicated that disrupting the microbiota with streptomycin treatment was insufficient to permit shedding of the systemic strain, as the newly available resources were likely immediately utilized by established intestinal *Salmonella*. Furthermore, since kanamycin does not enter mammalian cells, these results collectively indicate that established intestinal *Salmonella* occupy an extracellular transmission niche in the distal gut and exclude the bacteria that are reseeding the intestine from systemic sites.

### Early inhibitory priority effects in the gastrointestinal tract are facilitated by intraspecies competition for a nutritional niche

Our data show that intraspecies priority effects govern *Salmonella* population assembly in the distal gut. Since our previous results demonstrated that clonal expansion and priority effects in the cecum and colon could occur by 7 days post-infection (Figure 2, 7A), we hypothesized that nutrient acquisition was very important during this stage of colonization. Indeed, ecological theory has implicated competition for nutrients as an important determinant in priority effects and community structure [28].

*S.* Typhimurium hydrogenase (*hyb*) is a key mediator of cecal ecosystem invasion and is required to consume a microbiota-derived metabolite [30]. In the un-inflamed gut of conventional mice with complex microbiota, hydrogenase enzymes facilitate consumption of hydrogen (H_2_) intermediates in a SPI1- and SPI2-independent manner [30]. Similarly, we show here that Hyb is important for gut colonization and fecal shedding in 129SvJ mice with an intact conventional microbiota (Figure S12). To test the role of Hyb in intraspecies priority effects, co-infections were performed in which all mice were injected IP with 10^3^ wild-type (WT) SL1344 bacteria and one group of mice was co-inoculated orally with 10^8^ *ΔhybΔSPI1ΔSPI2* isogenic mutant *S.* Typhimurium while control mice were co-inoculated orally with 10^8^ WT SL1344-*kan^R^*. The *hyb* mutation was constructed in a *ΔSPI1ΔSPI2* background to assess the need for hydrogenase in the context of a non-inflamed gut. The relative levels of each strain in the feces were monitored over 15 days of co-infection (Figure 7B–C). Importantly, the *Salmonella* shed in the feces at 4 and 7 days contained systemic WT bacteria and by 15 days post-infection were entirely comprised of the systemic WT strain in mice that received *ΔhybΔSPI1ΔSPI2* orally (Figure 7B). Total levels of fecal *Salmonella* were significantly lower in the *ΔhybΔSPI1ΔSPI2* co-infection group compared to controls (Figure S13A), which corresponds to the decreased fecal shedding of *ΔhybΔSPI1ΔSPI2* mutants during single oral infections (Figure S12B). The strain compositions in the feces of these mice throughout infection indicated that the increase in total fecal CFU on day 15 reflected rapid reseeding and shedding of the systemic WT strain concomitant with declining levels of the oral *ΔhybΔSPI1ΔSPI2* strain (Figure 7C). Taken together, we have demonstrated that the hydrogenase mutant was unable to effectively invade the cecal and colonic niche (Figure 7D), thereby nullifying any priority effects and allowing systemic *Salmonella* to colonize the distal gut with subsequent transmission in feces (Figure 7C–D).

### *S.* Typhimurium SPI1 and SPI2 are required to maintain intraspecies colonization resistance during persistent infection

**Figure 8:**
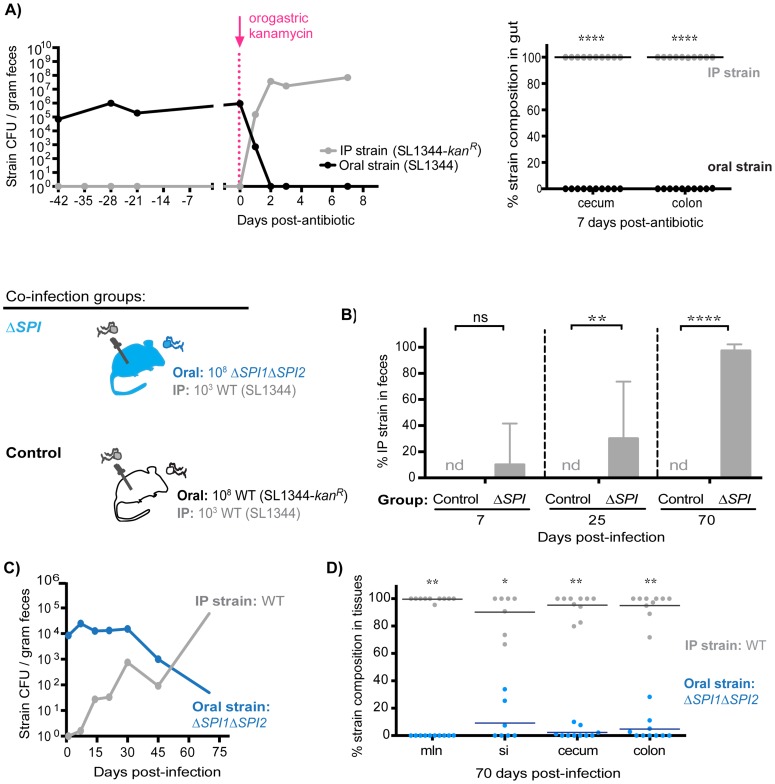
*S.* Typhimurium SPI1 and SPI2 are required to maintain intraspecies colonization resistance in the intestinal tract during persistent infection. A) Mice were co-infected with 10^8^ SL1344 orally and 10^3^ SL1344-*kan^R^* IP. A single dose of 20 mg kanamycin in 200 µl water was delivered by oral gavage 42 days post-infection. Data are representative of two independent experiments (n = 10). *Left:* Plot depicts geometric means of oral (black) and IP (gray) strains in feces. *Right:* Percent of *Salmonella* comprised of oral and IP strains in gut tissues 7 days after kanamycin treatment. Each circle represents an individual animal. ****p<0.0001, unpaired Mann-Whitney tests. B) All co-infected mice received 10^3^ WT SL1344 by IP injection (gray *Salmonella*). Mice in the control group received 10^8^ WT SL1344-*kan^R^* orally (white *Salmonella*), while mice in the ΔSPI group received 10^8^ Δ*SPI1*Δ*SPI2* orally (blue *Salmonella*). Data are representative of two independent experiments (n = 10/group). Percent of fecal *Salmonella* comprised of the IP strain (WT SL1344) at the indicated days post-infection. Limit of percent IP strain detection was 0.0011% by differential plating, nd = not detected. ns = not significant, **p = 0.0075, ****p<0.0001, unpaired Mann-Whitney tests. C) *S.* Typhimurium strain composition in feces of mice in the ΔSPI co-infection group. Geometric means of the Δ*SPI1*Δ*SPI2* (oral) and WT SL1344 (IP) strains shed in feces over 70 days of co-infection. D) Percent of *Salmonella* comprised of Δ*SPI1*Δ*SPI2* (oral) and WT SL1344 (IP) in intestinal tissues after 70 days of co-infection. mln = mLN, si = small intestine. Each circle represents an individual animal, lines at medians. Limit of detection was 0.1% by differential plating. *p = 0.0156, **p = 0.0020, Wilcoxon matched-pairs signed rank tests.

To determine whether intraspecies colonization resistance was still dependent on the maintenance of the extracellular intestinal niche during persistent infection, co-infected mice (oral: SL1344, IP: SL1344-*kan^R^*) were treated with a single dose of kanamycin 42 days post-infection. Within 24 hours of antibiotic administration, fecal shedding of the established intestinal SL1344 was decreased by ∼6 logs concomitant with a ∼5 log increase in the systemic SL1344-*kan^R^* strain (Figure 8A, *left*). The systemic strain was exclusively shed in the feces 2 days post-antibiotic treatment (Figure 8A, *left*), and comprised the entire *Salmonella* population in the cecum and colon after 7 days (Figure 8A, *right*). These data thus indicate that the extracellular niche in the cecum and colon is required to maintain intraspecies colonization resistance during persistent infection, which actively inhibits successful fecal shedding of systemic *Salmonella*.

To gain more insight into how *S.* Typhimurium competitively excludes incoming challengers from colonizing the distal gut niche, we tested the potential role of the key virulence factors *Salmonella* Pathogenicity Islands SPI1 and SPI2, which encode type III secretion systems that deliver effector proteins required for persistence in host tissues [32], [42]–[44] and fecal transmission [33]. Co-infections were performed in which mice simultaneously received 10^3^ WT SL1344 by IP and 10^8^ isogenic Δ*SPI1*Δ*SPI2* mutant bacteria orally. In the Δ*SPI1*Δ*SPI2* co-infected mice, the IP-injected WT bacteria were not present in significant numbers at day 7 (Figure 8B). However, by day 25, 21.43% of all fecal *Salmonella* were WT bacteria, and by day 70, 98.86% were WT *S.* Typhimurium (Figure 8B). In addition, the total fecal *Salmonella* CFU in the control (SL1344 oral, SL1344-*kan^R^* IP) and the Δ*SPI1*Δ*SPI2* co-infected mice were similar (Figure S13B), which is consistent with our result that the systemic WT strain reseeded and replicated within the intestinal tract once the Δ*SPI1*Δ*SPI2* mutant was cleared (Figure 8C). Indeed, examination of strain abundances in intestinal tissues after 70 days of co-infection confirmed that the systemic IP strain had predominantly colonized the mLN, small intestine, cecum, and colon while the initial Δ*SPI1*Δ*SPI2* mutant was cleared from these sites (Figure 8D). These studies demonstrate that SPI1 and SPI2 are required for the established intestinal *Salmonella* population to maintain active colonization resistance against systemic reseeding bacteria.

## Discussion

Microbial fecal shedding by chronically infected hosts is the major source of new infection and disease for many enteropathogenic microbes. However, very little is known about the dynamics of *Salmonella* subpopulations within mammalian hosts and what their relative contributions are to host-to-host transmission. Community assembly theory provides a framework for understanding infection processes, and in this study, we defined the *S.* Typhimurium metapopulation structure that arose during persistent infection. We then applied ecological principles that govern community assembly to determine the contribution of different *Salmonella* subpopulations to fecal shedding.

Our tagged strain approach revealed that distinct *S.* Typhimurium subpopulations arose within different host tissues, resulting in a metapopulation structure with variable migration between sites. After 35 days of infection, the WITS compositions between the liver and spleen closely matched each other, suggesting that robust migration pathways in the blood and lymphatics exist between these tissues. Previous studies of acute infection in susceptible C57BL/6 mice determined that hematogenous spread 48 hours post-infection resulted in *S.* Typhimurium mixing between the spleen and liver [23]. Expanding our WITS analyses to include a more comprehensive set of infected tissues, we determined that *Salmonella* in systemic sites were distinct from subpopulations in the intestinal tract. Interestingly, we found that the WITS profiles in the PP and small intestine were also dissimilar from those in the cecum and colon. This likely represents stochastic invasion of the PP by a subset of individual WITS strains, while different subsets initiate separate infection foci in other tissues. Indeed, a recent study of early infection dynamics determined that PP invasion fueled spread to the mLN while an independent pool of bacteria initiated splenic and hepatic infection [24]. Our work suggests that these initial colonization dynamics shape the metapopulation structure that arises and is maintained throughout persistent infection. Quantifying the differences in systemic, proximal and distal gut sites with Bray-Curtis dissimilarity scores, we were able to gain new insights into the importance of the distal gut as a transmission niche.

Surprisingly, we have shown that systemic *Salmonella* can only colonize the distal gut upon clearance of the established intestinal subpopulation with an oral kanamycin treatment. In contrast, treatment with streptomycin, to which the SL1344 strain is resistant, was insufficient to permit shedding of the systemic strain. This suggests that disrupting microbiota-mediated colonization resistance does not create new niches for systemic bacteria to colonize. Previous studies found that administration of ciprofloxacin killed extracellular *Salmonella* and permitted tolerant bacteria within dendritic cells of the cecal lymph node to colonize the cecum [45], [46]. Although this fluoroquinolone treatment also ablated systemic *Salmonella* [45], these studies all highlight the intensely competitive dynamics between *Salmonella* within the distal gut. Competition for gut colonization was also reported with *E. coli* K12 strains in germ-free mice, although differences in colonization ability was due to varying fitness costs of antibiotic resistances [47]. Our study with isogenic strains support the idea that intraspecies competition for nutrients excludes systemic bacteria from colonizing the distal gut, in which established *Salmonella* has saturated a required niche. Intraspecies priority effects have recently been described for commensal species of *Bacteroides* [48] and *E. coli* [49], but our findings with an enteropathogen that causes persistent systemic infection is novel. There may also be evidence of these competitive interactions during *Yersinia enterocolitica* microcolony formation within intestinal tissues, in which previously infected PP were less likely to be super-infected [21]. However, it remains unknown whether colonization can proceed if established *Yersinia* are eliminated. It is possible that this may be unique to *Salmonella* rather than a broad enteropathogen phenomenon, as this colonization resistance was not seen between isogenic *Campylobacter jejuni* strains in a transmission study involving chickens [50].

Host-adapted *Salmonella* serovars infect the gastrointestinal tract before disseminating to systemic sites such as the gallbladder, which has been classically thought to be the source of *Salmonella* transmitted in feces [51]. However, the contribution of systemic reseeding in the presence of an established *Salmonella* intestinal tract infection had never been investigated. We show that an established intestinal strain persisted in the cecum and colon, even when gallstone formation increased gallbladder levels of *S.* Typhimurium >10,000-fold. It is interesting to speculate that intraspecies colonization resistance may occur in other hosts that are persistently infected by *Salmonella*. For example, humans can carry S. Typhi for long periods of time possibly in the gallbladder [52]. Although gallbladder removal sometimes cures patients, over 20% of carriers continued to shed *S.* Typhi and *S.* Paratyphi in their stool [53], [54], which indicates an alternative persistent reservoir. While circulating *S.* Typhi in Kathmandu are resistant to nalidixic acid and several fluoroquinolones, patient gallbladder isolates are more sensitive to nalidixic acid, gatifloxacin, and ofloxacin, indicative of a limited role in typhoid transmission [55]. The relative contributions of systemic versus intestinal populations of *S.* Typhi to transmission are not known. Perhaps the presence of fecal “showers” of *S.* Typhi [4] are due to reseeding bacteria from systemic sites that gain access to spatial and nutritional resources in the gut. In our co-infection model, the oral strain comprised 88.83% of fecal *Salmonella* after 60 days, which was lower than the 97.96% observed after 30 days (p = 0.06, unpaired Mann-Whitney). Though this was not a significant difference, it is possible that the intestinal strain may lose its dominance at even later time points, at which point systemic *Salmonella* may reseed from mesenteric lymph node macrophages [31], [56], [57] and/or the gallbladder [1], [2], [10]. Intraspecies *Salmonella* colonization resistance could be shaping typhoid epidemiology in endemic regions, but future work is required to determine whether this occurs in other *Salmonella* serovars besides Typhimurium.

We have found that the clonal expansion of the intestinal subpopulation is responsible for increases in *S.* Typhimurium fecal shedding. The mechanisms by which this subpopulation expands and establishes intraspecies colonization resistance are likely multifactorial. *S.* Typhimurium fimbriae and adhesins are important for attachment to intestinal tissues [58]–[60] and may play a role in this intraspecies dynamic. Host immune responses contribute to *Salmonella* clearance [61]–[63], and could also be involved in influencing intraspecies colonization resistance. However, intraspecies colonization resistance was observed at 14 days post-infection and lasted over 102 days in the context of co-infections, cohousing experiments, and sequential infections. This suggests that neither the innate nor the adaptive immune responses alone could be responsible for the exclusion of systemic reseeding *Salmonella*.

Microbial communities undergo local diversification in different habitats within the host [12], [64]–[68], and we considered the possibility that genetic mutations could be responsible for *Salmonella* expansion and intraspecies colonization resistance. Previous studies with marked isogenic strains determined that spontaneous mutations alone do not shape *S.* Typhimurium colonization dynamics or fecal transmission during persistent infection in 129Sv mice. The dominance of a re-isolated strain was lost upon subsequent infection or passage in broth, and exhibited the same infectious dose (ID_50_) as a culture-grown strain [24], [33]. A study of systemic *S.* Typhimurium infection revealed that enhanced growth of bacteria were not due to the selection of mutants, but rather were transient phenotypic changes dependent on gene regulation [69]. Systemic *Salmonella* did not accumulate attenuating mutations during our experiments. This subpopulation adapted to the intestinal environment following ablation of the resident strain, and replicated to supershedder levels with rapid transmission to naïve mice. *Salmonella* transcriptional responses likely play an important role in expansion in the distal gut, and insight into these changes will elucidate other mechanisms by which priority effects are exerted.

Our studies with a hydrogenase mutant revealed that *Salmonella* competition for a microbiota-derived nutrient is one mechanism by which a challenging systemic strain is excluded from the distal gut transmission niche. According to the monopolization hypothesis, rapid population growth upon colonization of a new habitat results in the effective monopolization of resources, resulting in a strong inhibitory priority effect [70]. Since *Salmonella* are mainly localized in extracellular regions of the distal gut [33], it is tempting to speculate that other *Salmonella* factors required for nutrient acquisition play a role in intraspecies colonization resistance. The importance of nutrient acquisition in establishing priority effects could be applied to the development of novel therapies, in which targeting key metabolic pathways could potentially prevent pathogen colonization and transmission.

We have found that SPI1 and SPI2 contribute to intraspecies colonization resistance up to 70 days post-infection. Importantly, co-infected mice that received 10^8^ Δ*SPI1*Δ*SPI2* orally shed significant levels of WT systemic *Salmonella* beginning 25 days post-infection, with no significant changes in the total fecal shedding of *Salmonella*. This suggests that as soon as nutrient and/or spatial resources are made available by the clearance of the initial Δ*SPI1*Δ*SPI2* mutant, WT *Salmonella* spread from systemic tissues and rapidly expand within the intestinal tract. The T3SS encoded by these *Salmonella* pathogenicity islands deliver over thirty effectors with diverse functions [42]–[44], [71]. These effectors could act on *Salmonella* directly, or create an environment that kills strains reseeding from systemic tissues. These mechanisms could involve *Salmonella*-induced inflammation and modulation of the host immune response [72]. Inflammation also disrupts the host microbiota and allows the pathogen to metabolize newly available nutrients 1 [73]–[76]. Future work will seek to determine which of these are involved in establishing priority effects and exerting intraspecies colonization resistance.

Priority effects have long been known to shape community assembly in a variety of ecological systems, ranging from bacteria to larger eukaryotic organisms [27], [64]–[66], but this is the first time the phenomenon has been described for pathogen subpopulations during persistent infection within a host. In this landscape, the order in which *S.* Typhimurium arrive to the intestinal ecosystem dictates which bacteria are subsequently shed in the feces. The results presented herein demonstrate that colonization of distal gut tissues is a bottleneck for successful transmission, which subpopulations of *Salmonella* compete for. These studies may inform disease processes in host-adapted *Salmonella* serovars that cause invasive disease, yet are still transmitted fecal-orally. *S.* Typhimurium is a generalist pathogen that also infects livestock and humans, and thus our work has direct implications on public health [3], [4]. Our findings also highlight the potential for the application of ecological principles to epidemiology in order to predict dominant circulating strains during outbreaks. This work also sheds light on potential mechanisms that influence human-to-human transmission of non-typhoidal diarrheal infections, which can also be invasive in certain patients [5], [6]. A better understanding of these mechanisms might reveal novel therapeutic approaches, or even preventive measures in thwarting disease spread.

## Materials and Methods

### Ethics statement

Experiments involving animals were performed in accordance with NIH guidelines, the Animal Welfare Act, and US federal law. All animal experiments were approved by the Stanford University Administrative Panel on Laboratory Animal Care (APLAC) and overseen by the Institutional Animal Care and Use Committee (IACUC) under Protocol ID 12826. Animals were housed in a centralized research animal facility certified by the Association of Assessment and Accreditation of Laboratory Animal Care (AAALAC) International.

### Mouse strains and husbandry

129X1/SvJ and 129S1/SvImJ mice were obtained from Jackson Laboratories (Bar Harbor, ME). Male and female mice (5–7 weeks old) were housed under specific pathogen-free conditions in filter-top cages that were changed weekly by veterinary personnel. Sterile water and food were provided ad libitum. Mice were given 1 week to acclimate to the Stanford Research Animal Facility prior to experimentation.

### Bacterial strains and growth conditions

The *S.* Typhimurium strains used in this study were derived from the streptomycin-resistant parental strain SL1344 [77]. A missense mutation (*hisG46*) in SL1344 results in histidine auxotrophy [78]. The isogenic SL1344-*kan^R^* strain was created by replacing the *hisG* coding sequence with that of a kanamycin-resistance casette (*hisG::aphT*) using the methods of Datsenko and Wanner [33], [79]. Genetic manipulations were originally made in the *S.* Typhimurium LT2 background before being transferred to SL1344 by P22 transduction. This methodology was also used to construct wild-type isogenic tagged *Salmonella* (WITS strains: W1–W8), in which a unique 40-bp signature tag and the kanamycin-resistance cassette were inserted between the *malX* and *malY* pseudogenes. Grant *et. al.* previously established this approach and published the unique 40-bg sequence tags of 8 WITS strains [23], which were employed in this study (Table S1). Growth curves of W1–W8 in LB broth cultures were performed by optical density readings and plating for colony forming units (CFU) per milliliter (Figure S1A). *ΔSPI1ΔSPI2* (*orgA::tet, ssaV::kan*) was generated previously for use in other studies [80]. The *Δhyb* (*hypOhybABC::cm*) deletion was constructed as described by Maier *et. al.*, with P22 phage transduction to insert the deleted genomic region into the *ΔSPI-1ΔSPI-2* strain ([30], Table S1). All constructs were verified by PCR.

All *S.* Typhimurium strains were grown at 37°C with aeration in Luria-Bertani (LB) medium containing the appropriate antibiotics: streptomycin (200 µg/ml), kanamycin (40 µg/ml), tetracycline (15 µg/ml) and chloramphenicol (8 µg/ml). For mouse inoculation, an overnight culture of bacteria was spun down and washed with phosphate-buffered saline (PBS) before resuspension to obtain the desired concentration.

### Mouse infections

Food was removed 16 hours prior to all mouse infections. In WITS experiments, mice were inoculated with an equal mixture of strains W1–W8 via oral gavage of 10^8^ CFU in 100 µl PBS. For intraperitoneal (IP) infections, mice were injected with 10^3^ CFU in 100 µl PBS as previously described [32]. In the co-infection model, mice drank an oral dose of 10^8^ SL1344 in 20 µl PBS, then received an IP injection of 10^3^ SL1344-*kan^R^* immediately afterwards. Co-infection experiments were repeated using the reciprocal combination of strains, SL1344-*kan^R^* (oral) and SL1344 (IP), which had no effect on the trends observed.

### Monitoring fecal shedding of *S.* Typhimurium

Individual mice were identified by distinct tail markings and tracked throughout the duration of infection. Between 2–3 fresh fecal pellets were collected directly into eppendorf tubes and weighed at the indicated time points. Pellets were resuspended in 500 µl PBS and CFU/gram feces were determined by plating serial dilutions on LB agar plates with the appropriate antibiotics. Low (<10^4^ CFU/gram), moderate (<10^8^ CFU/gram), and super shedder (≥10^8^ CFU/gram) mice were identified based on previously established criteria [33], [37].

### *S.* Typhimurium burden in blood and tissues

Following collection of fresh fecal pellets, animals were sacrificed at the specified time points. Blood was collected by cardiac puncture and animals were euthanized by cervical dislocation. Sterile dissection tools were used to isolate individual organs, which were weighed prior to homogenization. The entire gastrointestinal tract was removed, and the small intestine was immediately separated from the distal gut and transferred to a new sterile petri dish. Visible PP (3–6/mouse) were isolated from the small intestine using sterile fine-tip straight tweezers and scalpels. PP, mLN, spleens, livers, and gall bladders were collected in 1 ml PBS. The small intestine, cecum, and colon were collected in 3 ml PBS. Homogenates were then serially diluted and plated onto LB agar containing the appropriate antibiotics to enumerate CFU/gram tissue. For co-infections with SL1344 and SL1344-*kan^R^*, several dilutions were plated to ensure adequate colonies (>100 CFU per sample) for subsequent patch plating to determine strain abundance.

### Genomic DNA extraction and WITS qPCR

For WITS experiments, 300 µl of tissue homogenate was inoculated into LB broth containing streptomycin (200 µg/ml) and kanamycin (40 µg/ml) as a recovery method to enrich for low abundance strains. An UltroSpec 2100pro spectrophotometer (Amersham Biosciences, Piscataway, NJ) was used to obtain optical density readings of the resulting bacterial cultures. Genomic DNA (gDNA) was extracted from 2×10^9^ *S.* Typhimurium from each sample in duplicate using a DNeasy blood and tissue kit (Qiagen, 69506) as per the manufacturer's protocol for Gram-negative bacteria.

All qPCRs were performed on an Applied Biosystems 7300 real-time PCR system. A 25 µl reaction contained 12.5 µl of FastStart SYBR Green Master Mix with Rox (Roche, 04913914001), 8 µl DNase/RNase-free water, 0.75 µl of forward and reverse (10 µM) primers (Table S1), and 3 µl of gDNA (1–10 ng). Standard curves were generated using gDNA from each W1–W8 strain. Reaction conditions were 50°C for 2 min; 95°C for 10 min; 40 cycles of 95°C for 15 s and 60°C for 1 min; followed by a dissociation stage of 95°C for 15 s, 60°C for 1 min, 95°C for 15 s, and 60°C for 15 s.

### Determining relative abundance of strains

To determine presence of a WITS strain, the qPCR value had to be above a minimum threshold value. This measure of primer specificity was determined by a negative control matrix, in which a specific primer pair was tested on ∼11.25 ng of non-template gDNA from each of the other 7 WITS strains. To test primer sensitivity, detection limits were determined by test plates containing known CFU of each strain. Briefly, colonies were washed off the plates with PBS and gDNA was extracted from plates with varying abundances of WITS (i.e. 1 CFU Strain A with 10^3^–10^5^ CFU Strain B). qPCR was performed and revealed a detection limit of 1 CFU/strain amidst over 4800 CFU from non-target strains. To verify that our method of broth recovery and qPCR analyses accurately rendered WITS abundances, we compared relative abundances of an equal mixture of culture-grown W1–W8 as determined by our qPCR strategy versus plating CFU of individual dilutions of each strain (Fig. S1B).

Plating onto selective LB agar containing streptomycin (20 µg/ml) and kanamycin (40 µg/ml) was used to determine strain abundances in co-infections, sequential challenges, and transmission experiments. In addition to patch plating a minimum of 100 CFU per sample, undiluted samples were plated on selective plates to increase detection limits. For super shedder mice, this permitted detection of a strain comprising just 0.00000001% of the total *S.* Typhimurium population.

### Determining equal fitness of WITS *in vivo*

The strain relative abundances were determined for each tissue in all of the 19 mice infected with the 10^8^ equal mixture of 8 WITS. The relative abundances of each WITS strain were analyzed by one-way ANOVA (parametric) and Kruskal-Wallis (non-parametric) tests in Prism statistical software. These analyses were performed for each tissue collected from infected mice. Non-significant P values indicated that a particular WITS was not under or over represented in any tissue type (Table S2). To further verify that certain WITS strains were not preferentially selected for, a control experiment was performed in which mice were orally infected with an inoculum comprised of a skewed WITS mixture (Figure S2A). Underrepresented strains: W2, W3, W5, W6 (4.17%–7.32% of inoculum), overrepresented strains: W1, W4, W7, W8 (17.39%–20.94% of inoculum). Relative abundances of WITS in infected tissues were determined by qPCR after 35 days of infection. For each of the 8 WITS, defined bins were constructed for a range of strain relative abundances, with which the observed frequencies were used to generate histograms (Figure S2B).

### Bray-Curtis dissimilarity analyses of WITS relative abundances in mouse tissues

Bray-Curtis dissimilarity scores were computed to quantitatively compare *Salmonella* population compositions in different sites. The relative abundance (*y*) of each WITS (*n*) was compared between two tissue sites *i* and *j*. The Bray-Curtis dissimilarity (d*^BCD^*) was calculated by:
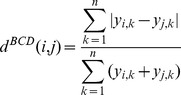
A value of 0 indicates an identical WITS composition between two sites, while a value of 1 signifies that two samples are completely dissimilar without any overlap in WITS representation.

### Gallstone model

The lithogenic diet established by Crawford *et. al.* ([38]) was modified in our experiments to include the normal rodent diet (Harlan, Teklad 2018) supplied in the Stanford Research Animal Facility. Mice were fed normal base chow supplemented with 1% cholesterol and 0.5% cholic acid (Harlan, Teklad custom research diet) for 10 weeks to induce cholesterol gallstone formation. Mice on control and lithogenic diets were anesthetized with isoflurane and shaved in the abdominal area for ultrasound imaging. A Vevo 2100 system (VisualSonics) was used to confirm gallstone formation. Mice were given 1 week to recover prior to infection with *S.* Typhimurium.

### Sequential infections with established and competing strains

For sequential infections in which the IP strain served as the initial strain, mice were first injected with 10^3^ SL1344 and the infection was allowed to establish for 35 days. Following that time period, mice were challenged with an oral dose of 10^8^ SL1344-*kan^R^*. In experiments with sequential oral infections, mice first received 10^8^ SL1344 orally by drinking. A persistent infection was allowed to establish for 102 days before oral challenge with 10^8^ SL1344-*kan^R^*. This sequential oral infection was performed with the reciprocal order of strains, in which SL1344-*kan^R^* was given as the initial strain and SL1344 given as the challenge strain.

### Cohousing experiments

Mice were infected orally with either 10^8^ SL1344 or SL1344-*kan^R^* and fecal shedding of *Salmonella* was monitored over 14 days prior to the start of the experiment. A SL1344-*kan^R^* super shedder donor was then cohoused with mice previously infected with SL1344, in addition to a naïve uninfected mouse as a control. Cohousing was continued for 10 days before the super shedder donor was removed. The reciprocal cohousing experiments were performed in which a SL1344 super shedder donor was cohoused with mice previously infected with SL1344-*kan^R^*.

### Kanamycin treatment

The aminoglycoside was administered orogastrically in a single dose of 20 mg (Sigma Aldrich, K4000) dissolved in 200 µl of water. Mice were transferred to new cages with autoclaved bedding, chow (Harlan, Teklad 2018S), and water at the time of administration.

### Statistical analyses

Prism (GraphPad) was used to create all figures and perform all statistical analyses. Intergroup comparisons of Bray-Curtis dissimilarity values (*e.g.* spleen-cecum versus colon-cecum) were analyzed by paired t-tests. Comparisons of oral and IP strain abundances within the same group of mice were evaluated with Wilcoxon matched-pairs signed rank tests. Differences in CFUs and strain composition between groups were examined by unpaired nonparametric Mann-Whitney tests. Significance was defined by p≤0.05.

## Supporting Information

Figure S1 **WITS enumeration and qPCR analysis strategy to determine relative abundances during infection.** Experimental design of WITS mouse infections and verification of strain quantification strategy. A) Growth curves of each strain in LB broth with appropriate antibiotics, no significant differences observed, validating our broth recovery approach. B) Percent strain composition of an equal mixture of culture-grown W1–W8 as determined by qPCR or plating of individual strain dilutions. No significant differences were observed. Results are representative of 3 independent experiments. C) WITS experimental design. Fecal samples were collected throughout the experiment, after which animals were sacrificed and tissues collected. Samples were plated on selective LB agar to enumerate total *S.* Typhimurium CFU and inoculated into selective LB broth in preparation for genomic DNA extraction. Quantitative PCR (qPCR) was performed to determine WITS abundances. D) Enumeration of WITS CFU in various host tissues by plating on LB agar containing kanamycin. Each circle represents an individual mouse (n = 19).
(TIF)

Figure S2 **Underrepresented WITS in the inoculum remain underrepresented in mouse tissues 35 days post-infection.** Mice were orally gavaged with a skewed inoculum in which strains W2, W3, W5, W6 were underrepresented and W1, W4, W7, and W8 were overrepresented (n = 4). *Pie chart:* skewed inoculum as determined by qPCR. A) Relative abundances of different WITS after 35 days of infection in the specified tissues; systemic tissues highlighted in gray. B) Number of observed frequencies of each WITS within each defined bin of relative abundances for all given tissues and mice. Overrepresented strains W1, W4, W7, and W8 (*top*) had increased observed frequencies of higher relative abundances compared to the underrepresented strains W2, W3, W5, and W6 (*bottom*), of which were either undetected or only had observed frequencies in the 0–0.2 range. The presence of small proportions of the underrepresented strains in systemic sites likely reflects the finding that a greater number of strains can disseminate to systemic tissues while the cecum and colon undergo a clonal expansion, a pattern that was observed in Figure 1.
(TIF)

Figure S3 **Intergroup analyses of Bray-Curtis dissimilarity scores in systemic and intestinal tissues.** Mice were orally infected with an equal mixture of 10^8^ WITS W1–W8 (n = 19, 3 independent experiments). After 35 days of infection, WITS relative abundances within various intestinal and systemic tissues were determined by qPCR. Bray-Curtis dissimilarity scores of the WITS composition between two organs were calculated (see Materials and Methods); score of 0 indicates identical WITS abundances, score of 1 indicates completely dissimilar WITS. Each circle represents an individual mouse (n = 19), lines represent medians. Intergroup differences were evaluated by paired t-tests. A) Intergroup comparisons between intestinal tissues: PP (pp), small intestine (si). **p = 0.0020, ***p<0.0003, ****p<0.0001. B) Intergroup comparisons between systemic organs and other tissues. *Top:* spleen, *** p = 0.0003, **** p<0.0001, *middle:* liver *** p = 0.0007 **** p<0.0001, *bottom:* gallbladder (gb), all not significant.
(TIF)

Figure S4 **Similar** ***Salmonella*** **burdens in mice after single oral, single IP, and co-infections.** Comparison of *Salmonella* CFU levels in co-infected mice versus single oral and single IP infections. Similar results were obtained with both SL1344 and SL1344-*kan^R^*, results from both are included. Mice were infected with one of the following: single oral infection with 10^8^ CFU (black), single IP infection with 10^3^ CFU (gray), or co-infection with 10^8^ orally and 10^3^ IP simultaneously (pink). Data represent two independent experiments. A) *Salmonella* CFU shed per gram feces in single oral (n = 20) and single IP infections (n = 24). *Left:* Geometric means of *S.* Typhimurium shed in feces over 25 days of infection, dashed line indicates limit of detection. *Right:* Individual shedding levels of mice after 25 days of infection, line at geometric mean. B) *Salmonella* CFU per gram of tissue for all infection routes (n = 8/group), line indicates mean. *Salmonella* burden was determined in systemic tissues: mesenteric lymph node (mLN), spleen, liver, and gallbladder as well as C) gut tissues: small intestine (s.i.), cecum, and feces.
(TIF)

Figure S5 **Established oral strains remain dominant in the distal gut after 60 days of co-infection.** Mice were co-infected as described in Figures 3–4 and monitored for 60 days. A) *Left:* Oral strain CFU (black) are plotted on the top half of the graph and the IP strain CFU (gray) on the bottom (median, range). Limit of detection for a single fecal sample is 10 CFU/gram feces. *Right:* Oral (black) and IP (gray) strain composition of *Salmonella* shed in feces after 60 days of co-infection (mean, SD). B) Animals were euthanized and the specified tissues were plated to determine of strain composition. Data are representative of two independent experiments (n = 20). Means with SD are depicted for oral (black) and IP (gray) strains. ns = non-significant, *p = 0.0166, **p = 0.0078, ***p<0.0007, ****p<0.0001, Wilcoxon matched-pairs signed rank tests.
(TIF)

Figure S6 **Confirmation of cholesterol gallstone formation.** A) Gallbladder ultrasound imaging of mice fed a 10-week control (top) or lithogenic diet (bottom). Blue dashed lines outline radiolucent gallbladders, the yellow arrow indicates a radiopaque gallstone. B) Lithogenic diet-induced gallstone formation (green asterisks) in the gallbladder of a 129X1/SvJ mouse.
(TIF)

Figure S7 **Gallstone formation increases reseeding of the gut by systemic** ***Salmonella*** **.** Control mice (black, n = 10) and mice with gallstones (green, n = 7) were infected with 10^3^ SL1344 by IP injection. Fecal *Salmonella* CFU were enumerated after 1, 7, and 14 days of infection, lines indicate geometric means. Data are representative of two independent experiments.
(TIF)

Figure S8 **In the presence of an established intestinal strain, challenging** ***Salmonella*** **are cleared from systemic and intestinal tissues.** Mice from sequential infections performed in Figure 6. SL1344 was used as the initial strain and SL1344-*kan^R^* was used as the challenging strain. Animals were sacrificed at the indicated time points and strain CFU were enumerated in tissues. Challenging strains (gray) were not detected. Limit of detection was determined by both differential plating and patch plating 100 CFU onto antibiotics; indicated by gray dashed lines. A) Mice were infected IP with 10^3^ SL1344 (Strain 1, black) for 35 days, followed by oral challenge with 10^8^ SL1344-*kan^R^* (Strain 2, gray). Animals were sacrificed 7 days post-challenge (42 dpi), n = 10. B) Mice were orally infected with 10^8^ SL1344 (Strain 1, black) and challenged orally with 10^8^ SL1344-*kan^R^* (Strain 2, gray) after 102 days. Animals were sacrificed 35 days post-challenge (137 dpi), n = 10. C) Recipient mice were orally infected with 10^8^ SL1344 for 14 days before co-housing with a supershedder SL1344-*kan^R^* donor. Animals were sacrificed 10 days post-cohousing. Established (SL1344, black) and donor (SL1344-*kan^R^*, gray) strain CFU were enumerated, n = 8.
(TIF)

Figure S9 **Established strains are resistant to super-colonization, and clearance of the challenging strain occurs more rapidly in super shedders.** A) Reciprocal order of strains from those used in sequential oral infections in Figure 6B. Mice were first inoculated with 10^8^ SL1344-*kan^R^* (gray) by drinking, which established a persistent infection for 60 days. Animals were subsequently challenged with 10^8^ SL1344 (black) by drinking. *Left:* Fecal CFU (mean, SD) of established strain SL1344-*kan^R^* and challenge strain SL1344 in feces. *Right:* Geometric mean of strain CFU in feces. Data are representative of two separate experiments (n = 6). B) Analysis of mice orally infected by sequential initial and challenge strains (described in above and in Figure 6B) based on prior shedding status. Percent abundance of the initial strain shed in feces in low (LS, n = 12), moderate (MS, n = 26), and super (SS, n = 8) shedder mice was determined for the specified days post-challenge. *p<0.05, ***p<0.001, ****p<0.0001, unpaired Mann-Whitney tests.
(TIF)

Figure S10 ***Salmonella*** **SL1344-** ***kan^R^*** **can be rapidly transmitted to naïve mice, and establishes a persistent intestinal infection exerting intraspecies colonization resistance against SL1344 from an infected donor.** A) Super shedder donors rapidly transmit SL1344-*kan^R^* to naïve uninfected mice. 14 days prior to cohousing, potential donor mice were infected orally with 10^8^ SL1344-*kan^R^*. Fecal *Salmonella* CFU/gram were tracked and a super shedder donor (gray asterisk) was identified, then cohoused with a recipient naïve mouse for 24 hours. Fecal shedding levels of SL1344-*kan^R^* from the recipient mice (open circles) were then tracked over 21 days. Data are representative of two independent experiments (n = 2 donors, 2 naïve recipients). B) Reciprocal order of strains from those used in cohousing experiments in Figure 6C. Mice were first infected orally with 10^8^ of either SL1344 or SL1344-*kan^R^* 14 days prior to cohousing. A SL1344 super shedder donor (black asterisk) was cohoused with SL1344-*kan^R^* infected recipient mice and removed after 18 days. Geometric means of *Salmonella* CFU/gram feces in recipient mice shedding the established SL1344-*kan^R^* strain (gray) or challenging donor SL1344 strain (black). Data are representative of two independent experiments (n = 2 donors, 6 recipients).
(TIF)

Figure S11 **Disruption of microbiota-mediated colonization resistance with streptomycin increases fecal shedding of intestinal** ***Salmonella*** **, but does not permit reseeding by the systemic strain.** Mice were co-infected with 10^8^ SL1344 orally and 10^3^ SL1344-*kan^R^* IP. A single dose of 5 mg streptomycin in 100 µl water was delivered by oral gavage after 30 days of co-infection (n = 5). Geometric means of oral (black) and IP (gray) strain CFU shed per gram feces. Limit of detection is 10 CFU/gram feces.
(TIF)

Figure S12 **A** ***Salmonella*** **hydrogenase mutant is cleared from feces and tissues after 15 days of infection.** Single oral infections were carried out in mice with 10^8^ WT SL1344-*kan^R^* (black), *ΔSPI1ΔSPI2* (blue), or *ΔhybΔSPI1ΔSPI2* (red). Data are representative of two independent experiments (n = 6/group). A) *Salmonella* CFU in mouse tissues after single oral infections with either WT or *ΔhybΔSPI1ΔSPI2* (red) after 15 days of infection. Each circle represents an individual mouse, lines at means. *p = 0.0152, **p = 0.002, unpaired Mann-Whitney tests. B) Fecal shedding of *Salmonella* was monitored at the specified time points post-infection (mean, SD). No significant differences in *Salmonella* CFU/gram feces were observed between WT and *ΔSPI1ΔSP-2* oral infections. *p<0.0411, **p<0.0022, unpaired Mann-Whitney tests.
(TIF)

Figure S13 **Total** ***Salmonella*** **in feces of mice co-infected with mutant strains.** A) Mice in the Δ*hyb* group received 10^8^ *ΔhybΔSPI1*Δ*SPI2* orally and 10^3^ WT SL1344 by IP. Control mice received 10^8^ WT SL1344-*kan^R^* orally and 10^3^ WT SL1344 by IP. Data are representative of two independent experiments (control n = 10, Δhyb n = 7). Total *Salmonella* CFU per gram feces, comprised of both oral and IP strains, detected over 15 days of co-infection for both control and Δhyb mouse groups (mean, SD). Comparison of total fecal *Salmonella* CFU between day 7 and day 15 in the Δhyb co-infected group is displayed in red (*p = 0.0373). *p_day4_ = 0.00, *p_day7_ = 0.0247, p*** = 0.0004, unpaired Mann-Whitney tests. B) Mice in the ΔSPI group received 10^8^ Δ*SPI1*Δ*SPI2* orally and 10^3^ WT SL1344 by IP. Control mice received 10^8^ WT SL1344-*kan^R^* orally and 10^3^ WT SL1344 by IP. Data are representative of two independent experiments (n = 10/group). Total *Salmonella* CFU per gram feces, comprised of both oral and IP strains, detected at the specified time points throughout 70 days of co-infection for both control and ΔSPI mouse groups (mean, SD). ns = not significant, unpaired Mann-Whitney tests.
(TIF)

Table S1 **Oligonucleotides used in this study.** Primers for tag insertion into SL1344 to generate WITS strains W1–8 and qPCR determination of WITS relative abundances. Previously published primers for Δ*hyb* deletion are referenced.
(XLSX)

Table S2 **Statistical analyses of WITS abundances in all sites sampled.** qPCR analyses were performed on all specified tissues collected from all mice (n = 19) to determine WITS relative abundances. The relative abundance of each WITS strain in each tissue was analyzed by one-way ANOVA (MS = mean square, F = F statistic, DFn = degrees of freedom numerator, DFd = degrees of freedom denominator) and Kruskal-Wallis tests. Significance defined by p≤0.05.
(XLSX)

Table S3 **Total burden of** ***Salmonella*** **within intestinal tissues in the co-infection model.** Mice were co-infected with SL1344 and SL1344-*kan^R^*, one strain orally and the other by IP injection. Mice were sacrificed after 30 days of infection, and CFU per gram tissue of the oral strain was converted into total CFU within the entire small intestine, cecum, or colon.
(XLSX)

Table S4 **Diet-induced gallstones increase levels of** ***Salmonella*** **within systemic tissues during co-infection.** Mice were either fed a control or lithogenic gallstone-inducing diet for 11 weeks. Animals were then co-infected with 10^8^ SL1344 and 10^3^ SL1344-*kan^R^*. Blood, spleen, liver, and gallbladder tissues were collected after 30 days.
(XLSX)

## References

[1] Parry CM, Hien TT, Dougan G, White NJ, Farrar JJ (2002) Typhoid Fever. N Engl J Med 347: 1770–1782. 12456854 10.1056/NEJMra020201

[2] Levin MM, Black RE, Lanata C (1982) Precise Estimation of the Numbers of Chronic Carriers of Salmonella typhi in Santiago, Chile, an Endemic Area. J INFECT DIS 146: 724–726. 7142746 10.1093/infdis/146.6.724

[3] Feasey NA, Dougan G, Kingsley RA, Heyderman RS, Gordon MA (2012) Invasive non-typhoidal salmonella disease: an emerging and neglected tropical disease in Africa. The Lancet 379: 2489–2499. 10.1016/S0140-6736(11)61752-2 PMC3402672 22587967

[4] Gopinath S, Carden S, Monack D (2012) Shedding light on Salmonella carriers. Trends in Microbiology 20: 320–327. 22591832 10.1016/j.tim.2012.04.004

[5] Kingsley RA, Msefula CL, Thomson NR, Kariuki S, Holt KE, et al (2009) Epidemic multiple drug resistant Salmonella Typhimurium causing invasive disease in sub-Saharan Africa have a distinct genotype. Genome Research 19: 2279. 19901036 10.1101/gr.091017.109 PMC2792184

[6] MacLennan CA, Gilchrist JJ, Gordon MA, Cunningham AF, Cobbold M, et al (2010) Dysregulated Humoral Immunity to Nontyphoidal Salmonella in HIV-Infected African Adults. Science 328: 508–512. 20413503 10.1126/science.1180346 PMC3772309

[7] Buchwald DS, Blaser MJ (1984) A review of human salmonellosis: II. Duration of excretion following infection with nontyphi Salmonella. Rev Infect Dis 3: 345–56.. 10.1093/clinids/6.3.345 6377442

[8] Sirinavin S, Garner P (1996) Antibiotics for treating salmonella gut infections. Sirinavin S, editor Chichester, UK: John Wiley & Sons, Ltd. 10.1002/14651858.CD001167 10796610

[9] Monack DM, Mueller A, Falkow S (2004) Persistent bacterial infections: the interface of the pathogen and the host immune system. Nature Reviews Microbiology 2: 747–765. 15372085 10.1038/nrmicro955

[10] Gonzalez-Escobedo G, Marshall JM, Gunn JS (2010) Chronic and acute infection of the gall bladder by Salmonella Typhi: understanding the carrier state. Nature Reviews Microbiology 9: 9–14. 21113180 10.1038/nrmicro2490 PMC3255095

[11] Costello EK, Stagaman K, Dethlefsen Les, Bohannan BJM, Relman DA (2012) The Application of Ecological Theory Toward an Understanding of the Human Microbiome. Science 336: 1255–1261. 22674335 10.1126/science.1224203 PMC4208626

[12] Levin BR (1999) Population Biology, Evolution, and Infectious Disease: Convergence and Synthesis. Science 283: 806–809. 9933155 10.1126/science.283.5403.806

[13] Hanski I, Gaggiotti OE (2004) Metapopulation biology: past, present, and future. In: Hanski I and Gaggiotti OE, editors. Ecology, Genetics and Evolution of Metapopulations. Burlington: Elsevier Academic Press. pp 3–22.

[14] Gaggiotti OE, Hanski I (2004) Mechanisms of population extinction. In: Hanski I and Gaggiotti OE, editors. Ecology, Genetics and Evolution of Metapopulations. Burlington: Elsevier Academic Press. pp 337–366.

[15] Hanski I (1998) Metapopulation dynamics. Nature 396: 41–49.

[16] Ovaskainen O, Hanski I (2004) Metapopulation dynamics in highly fragmented landscapes. In: Hanski I and Gaggiotti OE, editors. Ecology, Genetics and Evolution of Metapopulations. Burlington: Elsevier Academic Press. pp 73–103.

[17] Thomas CD, Hanski I (2004) Metapopulation dynamics in changing environments: butterfly responses to habitat and climate change. In: Hanski I and Gaggiotti OE, editors. Ecology, Genetics and Evolution of Metapopulations. Burlington: Elsevier Academic Press. pp 489–514.

[18] Melton-Witt JA, Rafelski SM, Portnoy DA, Bakardjiev AI (2012) Oral Infection with Signature-Tagged Listeria monocytogenes Reveals Organ-Specific Growth and Dissemination Routes in Guinea Pigs. Infection and Immunity 80: 720–732. 22083714 10.1128/IAI.05958-11 PMC3264322

[19] Barnes PD, Bergman MA, Mecsas J, Isberg RR (2006) Yersinia pseudotuberculosis disseminates directly from a replicating bacterial pool in the intestine. Journal of Experimental Medicine 203: 1591–1601. 16754724 10.1084/jem.20060905 PMC2118325

[20] Walters MS, Lane MC, Vigil PD, Smith SN, Walk ST, et al (2011) Kinetics of Uropathogenic Escherichia coli Metapopulation Movement during Urinary Tract Infection. mBio 3: e00303–11–e00303–11. 10.1128/mBio.00303-11 PMC3273315 22318320

[21] Oellerich MF, Jacobi CA, Freund S, Niedung K, Bach A, et al (2007) Yersinia enterocolitica Infection of Mice Reveals Clonal Invasion and Abscess Formation. Infection and Immunity 75: 3802–3811. 17562774 10.1128/IAI.00419-07 PMC1951990

[22] Kaiser P, Slack E, Grant AJ, Hardt W-D, Regoes RR (2013) PLOS Pathogens: Lymph Node Colonization Dynamics after Oral Salmonella Typhimurium Infection in Mice. plospathogensorg 9: e1003532. 10.1371/journal.ppat.1003532 PMC3777876 24068916

[23] Grant AJ, Restif O, McKinley TJ, Sheppard M, Maskell D, et al (2008) PLOS Biology: Modelling within-Host Spatiotemporal Dynamics of Invasive Bacterial Disease. PLoS Biology 6: e74. 18399718 10.1371/journal.pbio.0060074 PMC2288627

[24] Lim CH, Voedisch S, Wahl B, Rouf SF, Geffers R, et al (2014) Independent Bottlenecks Characterize Colonization of Systemic Compartments and Gut Lymphoid Tissue by Salmonella. PLoS Pathog 10: e1004270. 25079958 10.1371/journal.ppat.1004270 PMC4117638

[25] Sutherland JP (1974) Multiple stable points in natural communities. The American Naturalist 108: 859–873.

[26] Connell JH, Slatyer RO (1977) Mechanisms of succession in natural communities and their role in community stability and organization. The American Naturalist 111.

[27] Shulman MJ, Ogden JC, Ebersole JP, McFarland WN, Miller SL, et al (1983) Priority Effects in the Recruitment of Juvenile Coral Reef Fishes. Ecology 64: 1508.

[28] Kardol P, Souza L, Classen AT (2012) Resource availability mediates the importance of priority effects in plant community assembly and ecosystem function. Oikos 122: 84–94.

[29] Jiang L, Tan J, Pu Z (2010) An Experimental Test of Darwin's Naturalization Hypothesis. Am Nat 175: 415–423. 20170339 10.1086/650720

[30] Maier L, Vyas R, Cordova CD, Lindsay H, Schmidt TSB, et al (2013) Microbiota-Derived Hydrogen Fuels Salmonella Typhimurium Invasion of the Gut Ecosystem. Cell Host & Microbe 14: 641–651. 24331462 10.1016/j.chom.2013.11.002

[31] Monack DM (2004) Salmonella typhimurium Persists within Macrophages in the Mesenteric Lymph Nodes of Chronically Infected Nramp1+/+ Mice and Can Be Reactivated by IFN Neutralization. Journal of Experimental Medicine 199: 231–241. 14734525 10.1084/jem.20031319 PMC2211772

[32] Lawley TD, Chan K, Thompson LJ, Kim CC, Govoni GR, et al (2006) Genome-Wide Screen for Salmonella Genes Required for Long-Term Systemic Infection of the Mouse. PLoS Pathog 2: e11. 16518469 10.1371/journal.ppat.0020011 PMC1383486

[33] Lawley TD, Bouley DM, Hoy YE, Gerke C, Relman DA, et al (2007) Host Transmission of Salmonella enterica Serovar Typhimurium Is Controlled by Virulence Factors and Indigenous Intestinal Microbiota. Infection and Immunity 76: 403–416. 17967858 10.1128/IAI.01189-07 PMC2223630

[34] Pedron T, Mulet C, Dauga C, Frangeul L, Chervaux C, et al (2012) A Crypt-Specific Core Microbiota Resides in the Mouse Colon. mBio 3: e00116–12–e00116–12. 22617141 10.1128/mBio.00116-12 PMC3372965

[35] Shade A, Jones SE, Caporaso JG, Handelsman J, Knight R, et al (2014) Conditionally Rare Taxa Disproportionately Contribute to Temporal Changes in Microbial Diversity. mBio 4: e01371–14 doi:–10.1128/mBio.01371–14 10.1128/mBio.01371-14 PMC4161262 25028427

[36] Bray JR, Curtis JT (1957) An ordination of the upland forest communities of southern Wisconsin. Ecological Monographs 27: 325–349.

[37] Gopinath S, Hotson A, Johns J, Nolan G, Monack D (2013) The Systemic Immune State of Super-shedder Mice Is Characterized by a Unique Neutrophil-dependent Blunting of TH1 Responses. PLoS Pathog 9: e1003408. 23754944 10.1371/journal.ppat.1003408 PMC3675027

[38] Crawford RW, Rosales-Reyes R, Ramirez-Aguilar MDLL, Chapa-Azuela O, Alpuche-Aranda C, et al (2010) Gallstones play a significant role in Salmonella spp. gallbladder colonization and carriage. Proceedings of the National Academy of Sciences 107: 4353–4358. 10.1073/pnas.1000862107 PMC2840110 20176950

[39] Endt K, Stecher B, Chaffron S, Slack E, Tchitchek N, et al (2010) The Microbiota Mediates Pathogen Clearance from the Gut Lumen after Non-Typhoidal Salmonella Diarrhea. PLoS Pathog 6: e1001097. 20844578 10.1371/journal.ppat.1001097 PMC2936549

[40] Bohnhoff M, Miller CP (1962) Enhanced Susceptibility to Salmonella Infection in Streptomycin-Treated Mice. J INFECT DIS 111: 117–127. 13968487 10.1093/infdis/111.2.117

[41] Barthel M, Hapfelmeier S, Quintanilla-Martinez L, Kremer M, Rohde M, et al (2003) Pretreatment of Mice with Streptomycin Provides a Salmonella enterica Serovar Typhimurium Colitis Model That Allows Analysis of Both Pathogen and Host. Infection and Immunity 71: 2839–2858. 12704158 10.1128/IAI.71.5.2839-2858.2003 PMC153285

[42] Galan JE (2001) Salmonella interactions with host cells: Type III Secretion at Work. Annual Review of Cell and Developmental Biology 17: 53–86. 10.1146/annurev.cellbio.17.1.53 11687484

[43] Coburn B, Li Y, Owen D, Vallance BA, Finlay BB (2005) Salmonella enterica Serovar Typhimurium Pathogenicity Island 2 Is Necessary for Complete Virulence in a Mouse Model of Infectious Enterocolitis. Infection and Immunity 73: 3219–3227. 15908346 10.1128/IAI.73.6.3219-3227.2005 PMC1111876

[44] McGhie EJ, Brawn LC, Hume PJ, Humphreys D, Koronakis V (2009) Salmonella takes control: effector-driven manipulation of the host. Current Opinion in Microbiology 12: 117–124. 19157959 10.1016/j.mib.2008.12.001 PMC2647982

[45] Kaiser P, Regoes RR, Dolowschiak T, Wotzka SY, Lengefeld J, et al (2014) Cecum Lymph Node Dendritic Cells Harbor Slow-Growing Bacteria Phenotypically Tolerant to Antibiotic Treatment. PLoS Biol 12: e1001793. 24558351 10.1371/journal.pbio.1001793 PMC3928039

[46] Diard M, Sellin ME, Dolowschiak T, Arnoldini M, Ackermann M, et al (2014) Antibiotic Treatment Selects for Cooperative Virulence of Salmonella Typhimurium. Current Biology 24: 2000–2005. 25131673 10.1016/j.cub.2014.07.028

[47] Onderdonk A, Marshall B, Cisneros R, Levy B (1981) Competition between congenic Escherichia coli K-12 strains in vivo. Infection and Immunity 1: 74. 10.1128/iai.32.1.74-79.1981 PMC350589 7012037

[48] Lee SM, Donaldson GP, Mikulski Z, Boyajian S, Ley K, et al (2013) Bacterial colonization factors control specificity and stability of the gut microbiota. Nature 501: 426–429. 23955152 10.1038/nature12447 PMC3893107

[49] Leatham MP, Banerjee S, Autieri SM, Mercado-Lubo R, Conway T, et al (2009) Precolonized Human Commensal Escherichia coli Strains Serve as a Barrier to E. coli O157:H7 Growth in the Streptomycin-Treated Mouse Intestine. Infect Immun 7: 2876–2886. 10.1128/IAI.00059-09 PMC2708557 19364832

[50] Grant AJ, Coward C, Jones MA, Woodall CA, Barrow PA, et al (2005) Signature-Tagged Transposon Mutagenesis Studies Demonstrate the Dynamic Nature of Cecal Colonization of 2-Week-Old Chickens by Campylobacter jejuni. Appl Environ Microbiol 71: 8031–8041. 16332783 10.1128/AEM.71.12.8031-8041.2005 PMC1317333

[51] Everest P, Wain J, Roberts M, Rook G, Dougan G (2001) The molecular mechanisms of severe typhoid fever. Trends in Microbiology 9: 316–320. 11435104 10.1016/s0966-842x(01)02067-4

[52] Vaishnavi C, Singh S, Kochhar R, Bhasin D, Singh G, et al (2005) Prevalence of Salmonella enterica serovar Typhi in bile and stool of patients with biliary disease and those requiring biliary drainage for other purposes. Japanese Journal of Infectious Disease 58: 363–365. 16377868

[53] Vogelsang TM, Boe J (1948) Temporary and chronic carriers of Salmonella typhi and Salmonella paratyphi B. The Journal of Hygiene 46: 252–261. 18122185 10.1017/s0022172400036378 PMC2235125

[54] Ristori C, Rodriguez H, Vicent P, Ferreccio C, Garcia J, et al (1982) Persistence of the Salmonella Typhi-Paratyphi carrier state after gallbladder removal. Bull Pan Am Health Organ 16: 361–366. 7165819

[55] Dongol S, Thompson CN, Clare S, Nga TVT, Duy PT, et al (2012) The Microbiological and Clinical Characteristics of Invasive Salmonella in Gallbladders from Cholecystectomy Patients in Kathmandu, Nepal. PLoS ONE 7: e47342. 23077595 10.1371/journal.pone.0047342 PMC3471863

[56] Goodpasture EW (1936) Concerning the pathogenesis of typhoid fever. American Journal of Pathology 13: 175–185. PMC1911110 19970319

[57] Eisele NE, Ruby T, Jacobson A, Manzanillo PS, Cox JS, et al (2013) Salmonella Require the Fatty Acid Regulator PPARδ for the Establishment of a Metabolic Environment Essential for Long-Term Persistence. Cell Host & Microbe 14: 171–182. 23954156 10.1016/j.chom.2013.07.010 PMC3785333

[58] Bäumler AJ, Tsolis RM, Bowe FA, Kusters JG, Hoffmann S, et al (1995) The pef fimbrial operon of Salmonella typhimurium mediates adhesion to murine small intestine and is necessary for fluid accumulation in the infant mouse. Infection and Immunity 64: 61–68. 10.1128/iai.64.1.61-68.1996 PMC173728 8557375

[59] Dorsey CW, Laarakker MC, Humphries AD, Weening EH, Bäumler AJ (2005) Salmonella enterica serotype Typhimurium MisL is an intestinal colonization factor that binds fibronectin. Molecular Microbiology 57: 196–211. 15948960 10.1111/j.1365-2958.2005.04666.x

[60] Misselwitz B, Barrett N, Kreibich S, Vonaesch P, Andritschke D, et al (2012) Near Surface Swimming of Salmonella Typhimurium Explains Target-Site Selection and Cooperative Invasion. PLoS Pathog 8: e1002810. 22911370 10.1371/journal.ppat.1002810 PMC3406100

[61] Griffin AJ, McSorley SJ (2011) Development of protective immunity to Salmonella, a mucosal pathogen with a systemic agenda. Mucosal Immunology 4: 371–382. 21307847 10.1038/mi.2011.2 PMC4084725

[62] Wijburg OLC (2006) Innate secretory antibodies protect against natural Salmonella typhimurium infection. Journal of Experimental Medicine 203: 21–26. 16390940 10.1084/jem.20052093 PMC2118088

[63] Broz P, Ohlson MB, Monack DM (2012) Innate immune response to Salmonella typhimurium, a model enteric pathogen. Gut Microbes 3: 62–70. 22198618 10.4161/gmic.19141 PMC3370950

[64] Peay KG, Belisle M, Fukami T (2011) Phylogenetic relatedness predicts priority effects in nectar yeast communities. Proceedings of the Royal Society Biological Sciences 279: 749–758. 21775330 10.1098/rspb.2011.1230 PMC3248732

[65] Tan J, Pu Z, Ryberg WA, Jiang L (2012) Species phylogenetic relatedness, priority effects, and ecosystem functioning. Ecology 93: 1164–1172. 22764502 10.1890/11-1557.1

[66] Fukami T, Beaumont HJE, Zhang X-X (2007) Immigration history controls diversification in experimental adaptive radiation. Nature Letters 446: 436–439. 10.1038/nature05629 17377582

[67] Hernandez SB, Cota I, Ducret A, Aussel L (2012) Adaptation and Preadaptation of Salmonella enterica to Bile. PLoS Genet 8: e1002459. 22275872 10.1371/journal.pgen.1002459 PMC3261920

[68] Diard M, Garcia V, Maier L, Remus-Emsermann MNP, Regoes RR, et al (2013) Stabilization of cooperative virulence by the expression of an avirulent phenotype. Nature 494: 353–356. 23426324 10.1038/nature11913

[69] Mastroeni P, Morgan FJE, McKinley TJ, Shawcroft E, Clare S, et al (2011) Enhanced Virulence of Salmonella enterica Serovar Typhimurium after Passage through Mice. Infection and Immunity 79: 636–643. 21098099 10.1128/IAI.00954-10 PMC3028859

[70] Meester LD, Gomez A, Okamura B, Schwenk K (2002) The Monopolization Hypothesis and the dispersal–gene flow paradox in aquatic organisms. Acta Oecologica 23: 121–135.

[71] Brown NF, Vallance BA, Coombes BK, Valdez Y, Coburn BA, et al (2005) Salmonella Pathogenicity Island 2 Is Expressed Prior to Penetrating the Intestine. PLoS Pathog 1: e32. 16304611 10.1371/journal.ppat.0010032 PMC1287911

[72] Barman M, Unold D, Shifley K, Amir E, Hung K, et al (2008) Enteric Salmonellosis Disrupts the Microbial Ecology of the Murine Gastrointestinal Tract. Infection and Immunity 76: 907–915. 18160481 10.1128/IAI.01432-07 PMC2258829

[73] Stecher B, Robbiani R, Walker AW, Westendorf AM, Barthel M, et al (2007) Salmonella enterica Serovar Typhimurium Exploits Inflammation to Compete with the Intestinal Microbiota. PLoS Biol 5: e244. 10.1371/journal.pbio.0050244 PMC1951780 17760501

[74] Thiennimitr P, Winter SE, Winter MG, Xavier MN, Tolstikov V, et al (2011) Intestinal inflammation allows Salmonella to use ethanolamine to compete with the microbiota. Proceedings of the National Academy of Sciences 108: 17480–17485. 10.1073/pnas.1107857108 PMC3198331 21969563

[75] Becker D, Selbach M, Rollenhagen C, Ballmaier M, Meyer TF, et al (2006) Robust Salmonella metabolism limits possibilities for new antimicrobials. Nature 440: 303–307. 16541065 10.1038/nature04616

[76] Kaiser BLD, Li J, Sanford JA, Kim Y-M, Kronewitter SR, et al (2013) A Multi-Omic View of Host-Pathogen-Commensal Interplay in Salmonella-Mediated Intestinal Infection. PLoS ONE 8: e67155. 23840608 10.1371/journal.pone.0067155 PMC3694140

[77] Smith BP, Reina-Guerra M, Joiseth SK, Stocker BA, Habasha F, et al (1984) Aromatic-dependent Salmonella typhimurium as modified live vaccines for calves. Am J Vet Res 1: 59–66. 6367561

[78] Henry T, García-del Portillo F, Gorvel JP (2005) Identification of Salmonella functions critical for bacterial cell division within eukaryotic cells. Molecular Microbiology 56: 252–267. 15773994 10.1111/j.1365-2958.2005.04540.x

[79] Datsenko KA, Wanner BL (2000) One-step inactivation of chromosomal genes in Escherichia coli K-12 using PCR products. Proceedings of the National Academy of Sciences 97: 6640–6645. 10.1073/pnas.120163297 PMC18686 10829079

[80] Broz P, Newton K, Lamkanfi M, Mariathasan S, Dixit VM, et al (2010) Redundant roles for inflammasome receptors NLRP3 and NLRC4 in host defense against Salmonella. J Exp Med 207: 1745–1755. 20603313 10.1084/jem.20100257 PMC2916133

