# Supplementary figures and images for: Intraspecies Competition for Niches in the Distal Gut Dictate Transmission during Persistent Salmonella Infection
Source: PLoS Pathog. 2014 Dec 4;10(12):e1004527. doi: 10.1371/journal.ppat.1004527 (PMC4256465; doi:10.1371/journal.ppat.1004527)

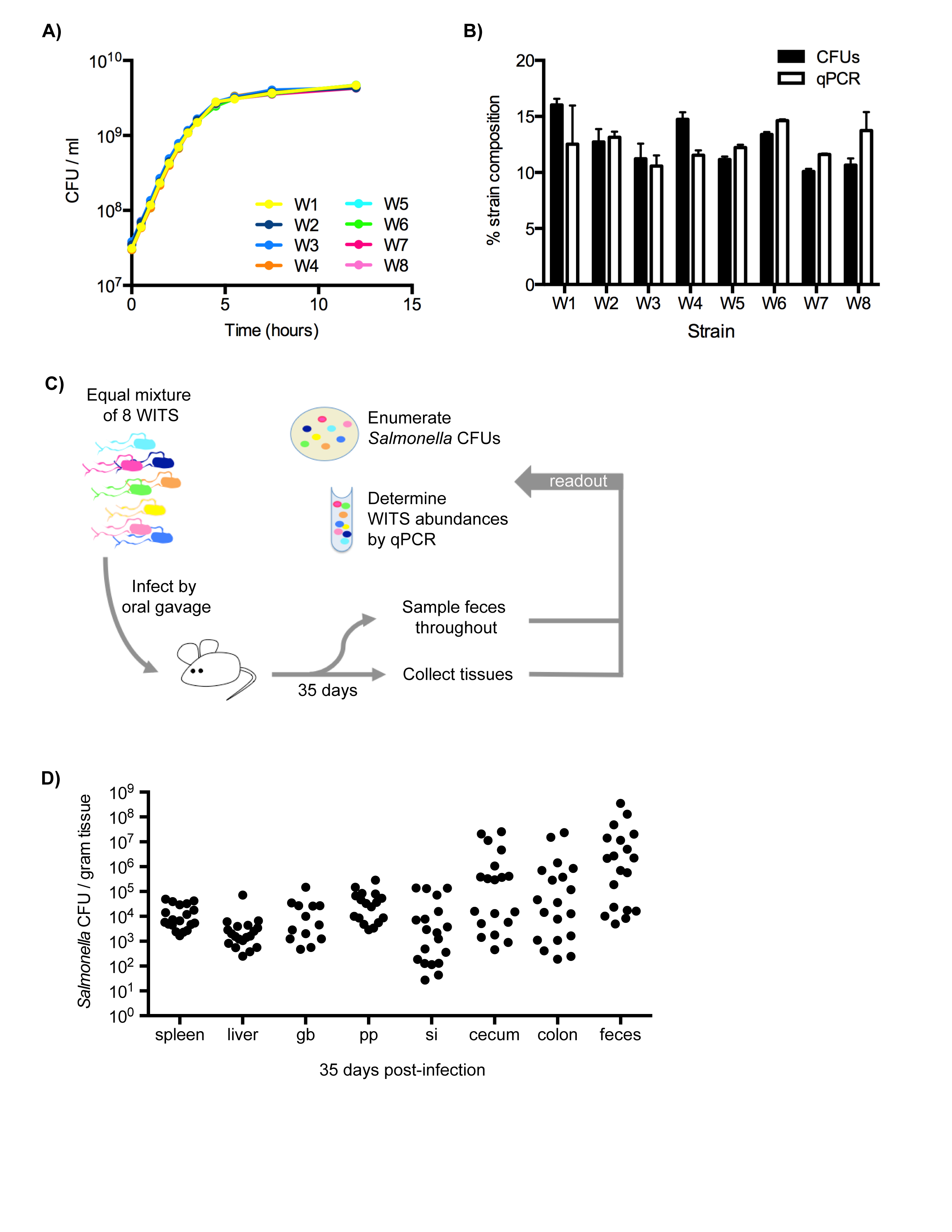

Supplement: Figure S1 — WITS enumeration and qPCR analysis strategy to determine relative abundances during infection. Experimental design of WITS mouse infections and verification of strain quantification strategy. A) Growth curves of each strain in LB broth with appropriate antibiotics, no significant differences observed, validating our broth recovery approach. B) Percent strain composition of an equal mixture of culture-grown W1–W8 as determined by qPCR or plating of individual strain dilutions. No significant differences were observed. Results are representative of 3 independent experiments. C) WITS experimental design. Fecal samples were collected throughout the experiment, after which animals were sacrificed and tissues collected. Samples were plated on selective LB agar to enumerate total S. Typhimurium CFU and inoculated into selective LB broth in preparation for genomic DNA extraction. Quantitative PCR (qPCR) was performed to determine WITS abundances. D) Enumeration of WITS CFU in various host tissues by plating on LB agar containing kanamycin. Each circle represents an individual mouse (n = 19). (TIF) [file ppat.1004527.s001.tif]

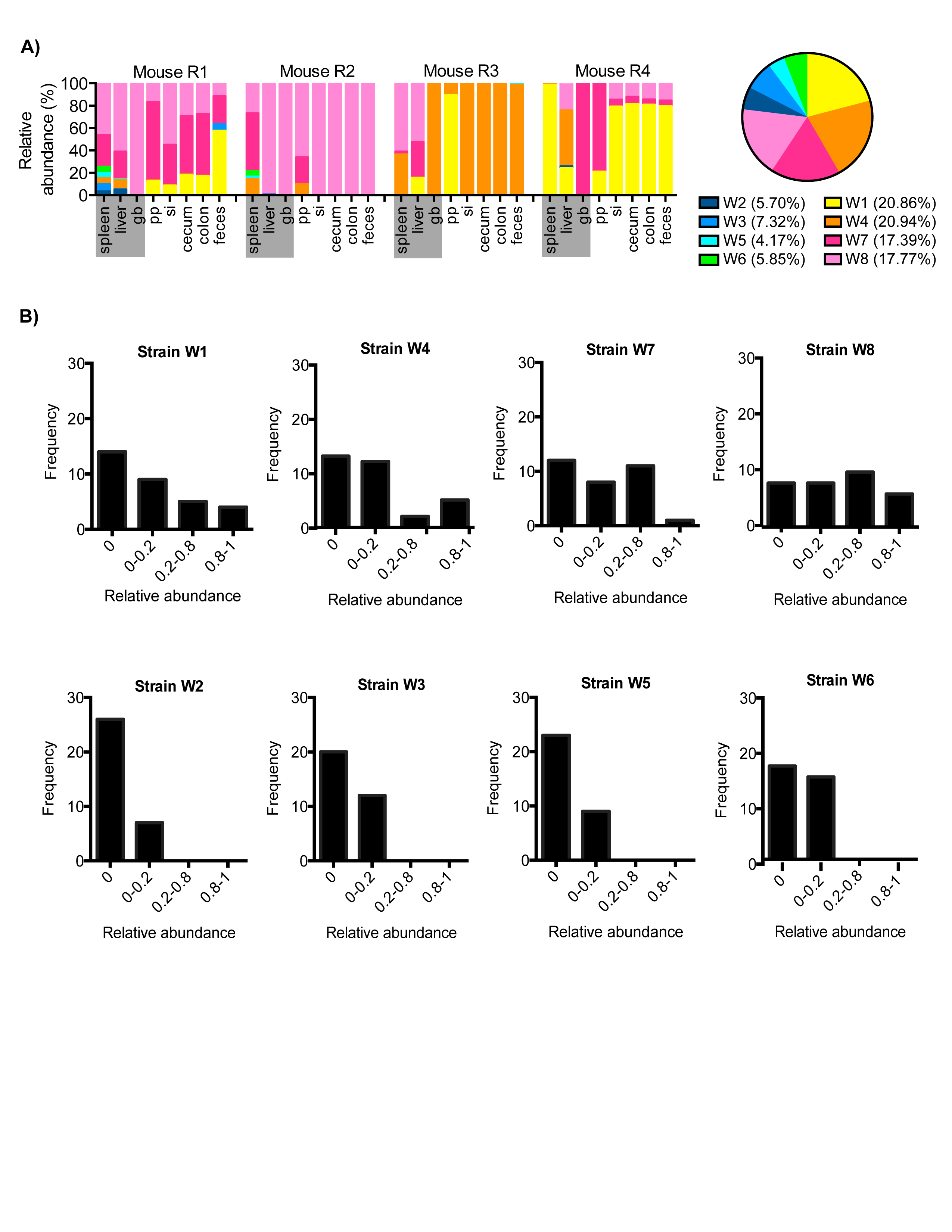

Supplement: Figure S2 — Underrepresented WITS in the inoculum remain underrepresented in mouse tissues 35 days post-infection. Mice were orally gavaged with a skewed inoculum in which strains W2, W3, W5, W6 were underrepresented and W1, W4, W7, and W8 were overrepresented (n = 4). Pie chart: skewed inoculum as determined by qPCR. A) Relative abundances of different WITS after 35 days of infection in the specified tissues; systemic tissues highlighted in gray. B) Number of observed frequencies of each WITS within each defined bin of relative abundances for all given tissues and mice. Overrepresented strains W1, W4, W7, and W8 (top) had increased observed frequencies of higher relative abundances compared to the underrepresented strains W2, W3, W5, and W6 (bottom), of which were either undetected or only had observed frequencies in the 0–0.2 range. The presence of small proportions of the underrepresented strains in systemic sites likely reflects the finding that a greater number of strains can disseminate to systemic tissues while the cecum and colon undergo a clonal expansion, a pattern that was observed in Figure 1. (TIF) [file ppat.1004527.s002.tif]

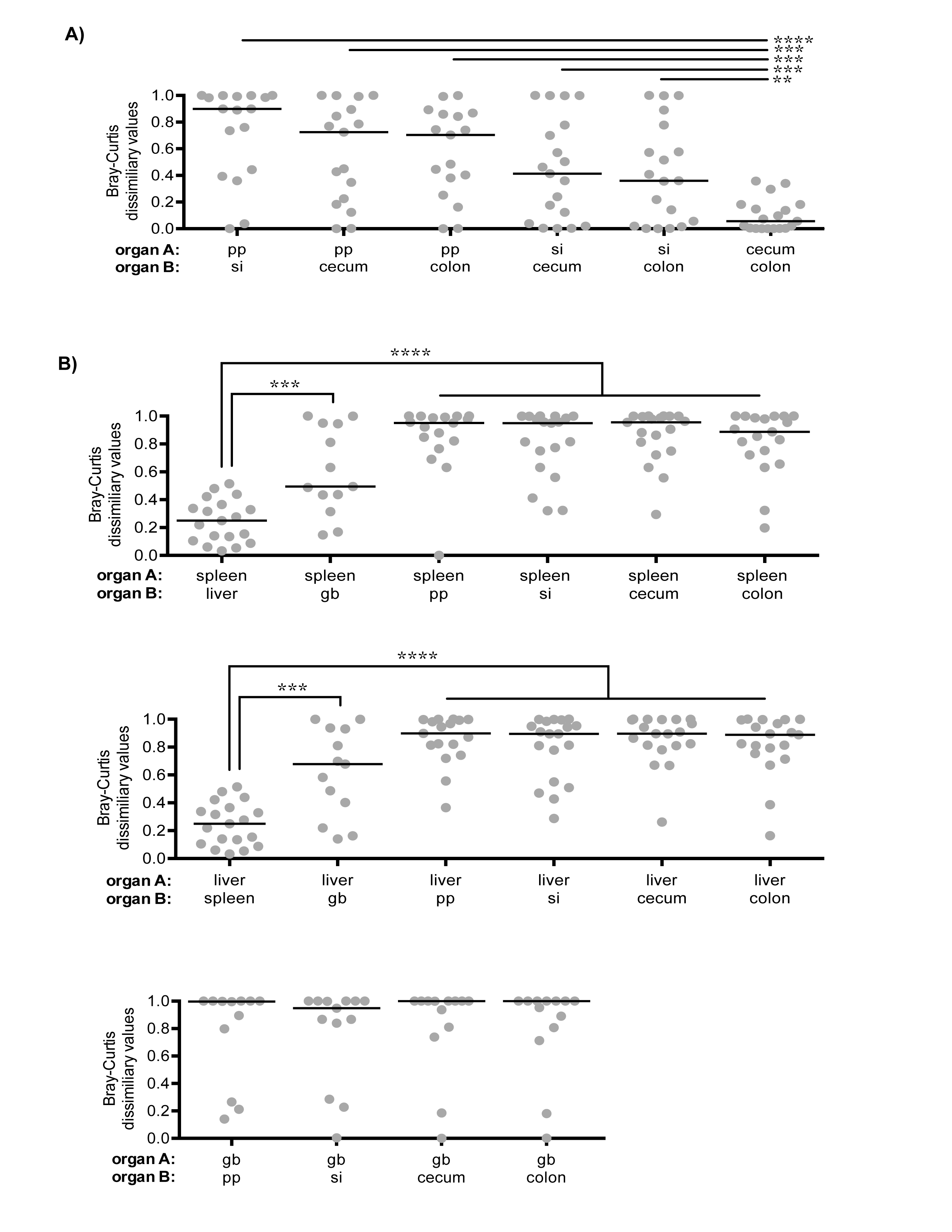

Supplement: Figure S3 — Intergroup analyses of Bray-Curtis dissimilarity scores in systemic and intestinal tissues. Mice were orally infected with an equal mixture of 108 WITS W1–W8 (n = 19, 3 independent experiments). After 35 days of infection, WITS relative abundances within various intestinal and systemic tissues were determined by qPCR. Bray-Curtis dissimilarity scores of the WITS composition between two organs were calculated (see Materials and Methods); score of 0 indicates identical WITS abundances, score of 1 indicates completely dissimilar WITS. Each circle represents an individual mouse (n = 19), lines represent medians. Intergroup differences were evaluated by paired t-tests. A) Intergroup comparisons between intestinal tissues: PP (pp), small intestine (si). **p = 0.0020, ***p<0.0003, ****p<0.0001. B) Intergroup comparisons between systemic organs and other tissues. Top: spleen, *** p = 0.0003, **** p<0.0001, middle: liver *** p = 0.0007 **** p<0.0001, bottom: gallbladder (gb), all not significant. (TIF) [file ppat.1004527.s003.tif]

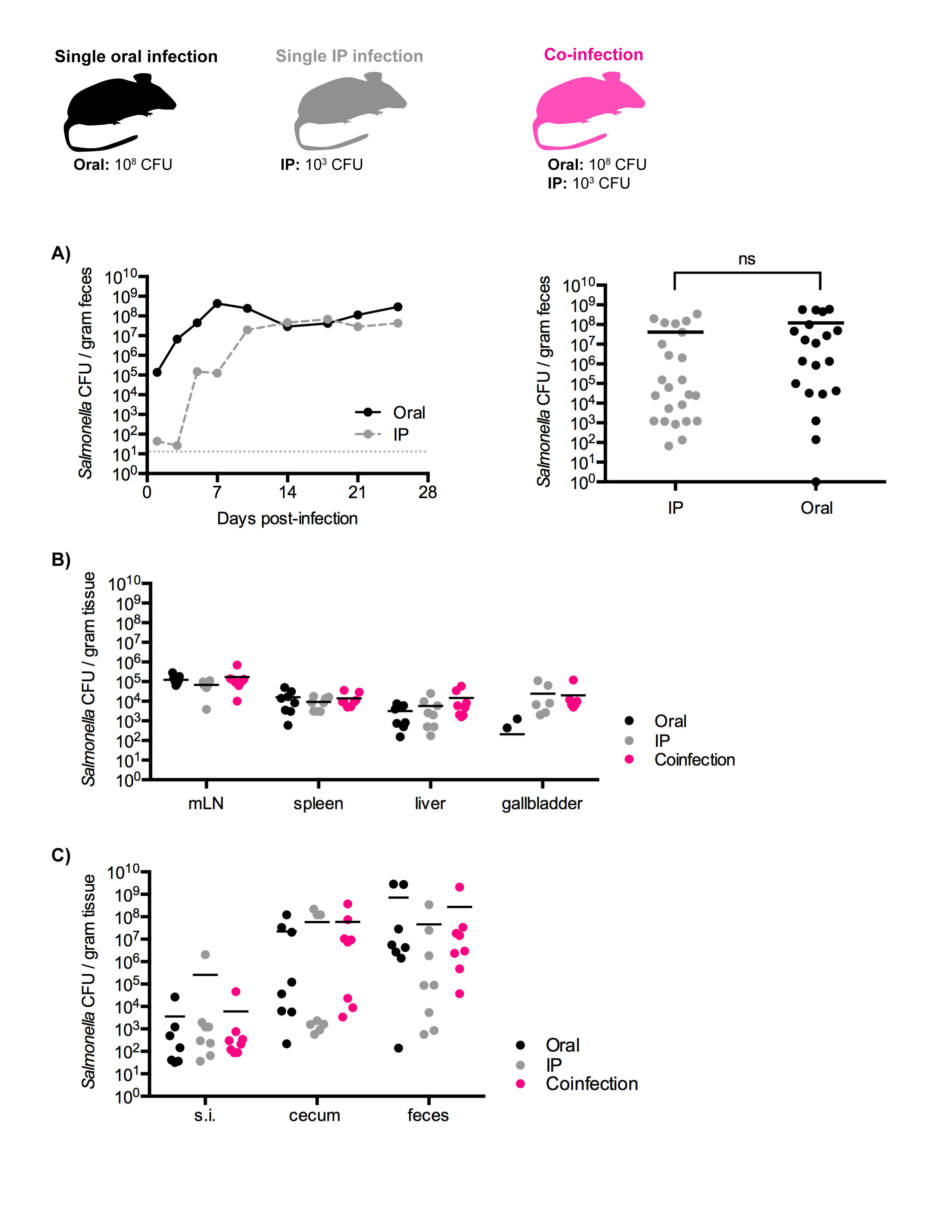

Supplement: Figure S4 — Similar Salmonella burdens in mice after single oral, single IP, and co-infections. Comparison of Salmonella CFU levels in co-infected mice versus single oral and single IP infections. Similar results were obtained with both SL1344 and SL1344-kanR, results from both are included. Mice were infected with one of the following: single oral infection with 108 CFU (black), single IP infection with 103 CFU (gray), or co-infection with 108 orally and 103 IP simultaneously (pink). Data represent two independent experiments. A) Salmonella CFU shed per gram feces in single oral (n = 20) and single IP infections (n = 24). Left: Geometric means of S. Typhimurium shed in feces over 25 days of infection, dashed line indicates limit of detection. Right: Individual shedding levels of mice after 25 days of infection, line at geometric mean. B) Salmonella CFU per gram of tissue for all infection routes (n = 8/group), line indicates mean. Salmonella burden was determined in systemic tissues: mesenteric lymph node (mLN), spleen, liver, and gallbladder as well as C) gut tissues: small intestine (s.i.), cecum, and feces. (TIF) [file ppat.1004527.s004.tif]

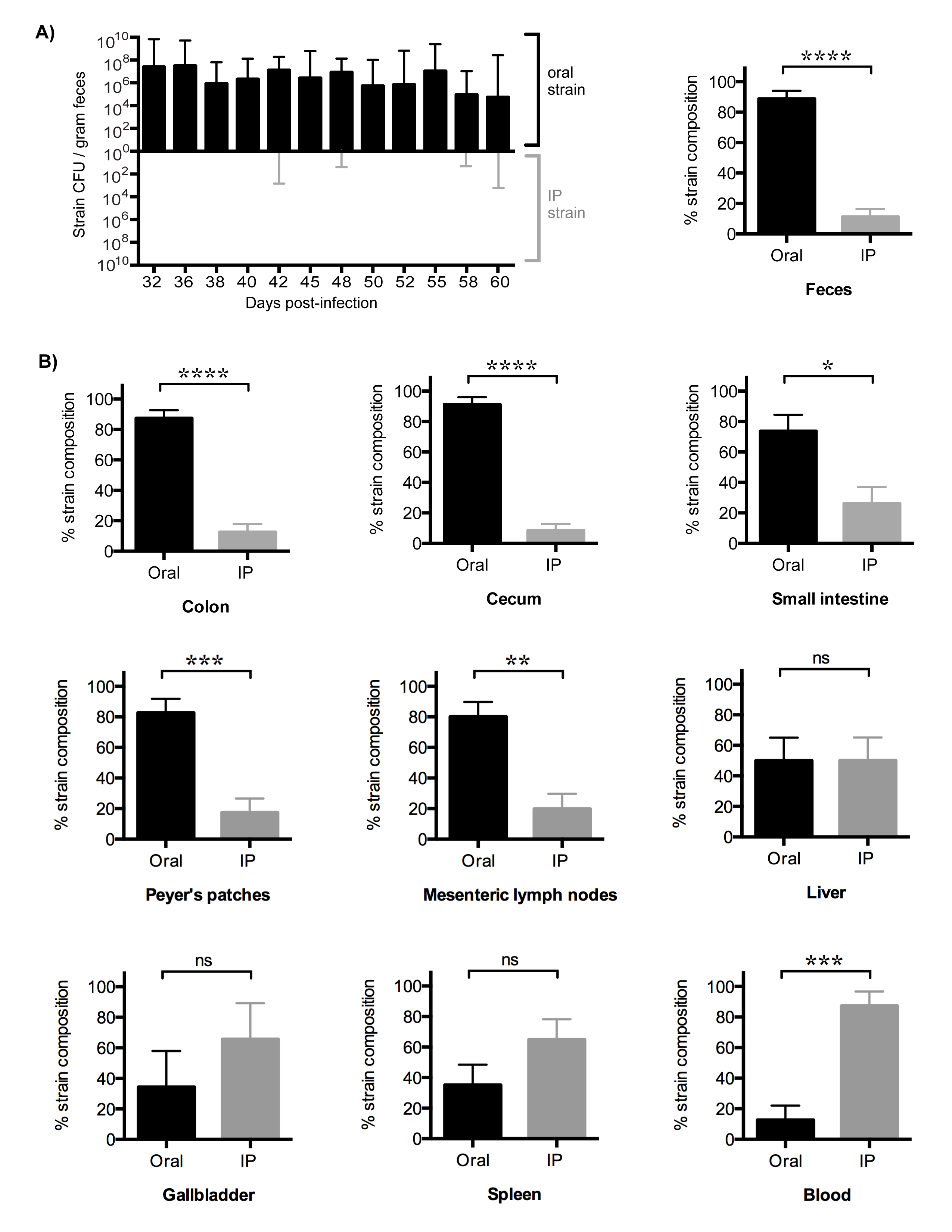

Supplement: Figure S5 — Established oral strains remain dominant in the distal gut after 60 days of co-infection. Mice were co-infected as described in Figures 3–4 and monitored for 60 days. A) Left: Oral strain CFU (black) are plotted on the top half of the graph and the IP strain CFU (gray) on the bottom (median, range). Limit of detection for a single fecal sample is 10 CFU/gram feces. Right: Oral (black) and IP (gray) strain composition of Salmonella shed in feces after 60 days of co-infection (mean, SD). B) Animals were euthanized and the specified tissues were plated to determine of strain composition. Data are representative of two independent experiments (n = 20). Means with SD are depicted for oral (black) and IP (gray) strains. ns = non-significant, *p = 0.0166, **p = 0.0078, ***p<0.0007, ****p<0.0001, Wilcoxon matched-pairs signed rank tests. (TIF) [file ppat.1004527.s005.tif]

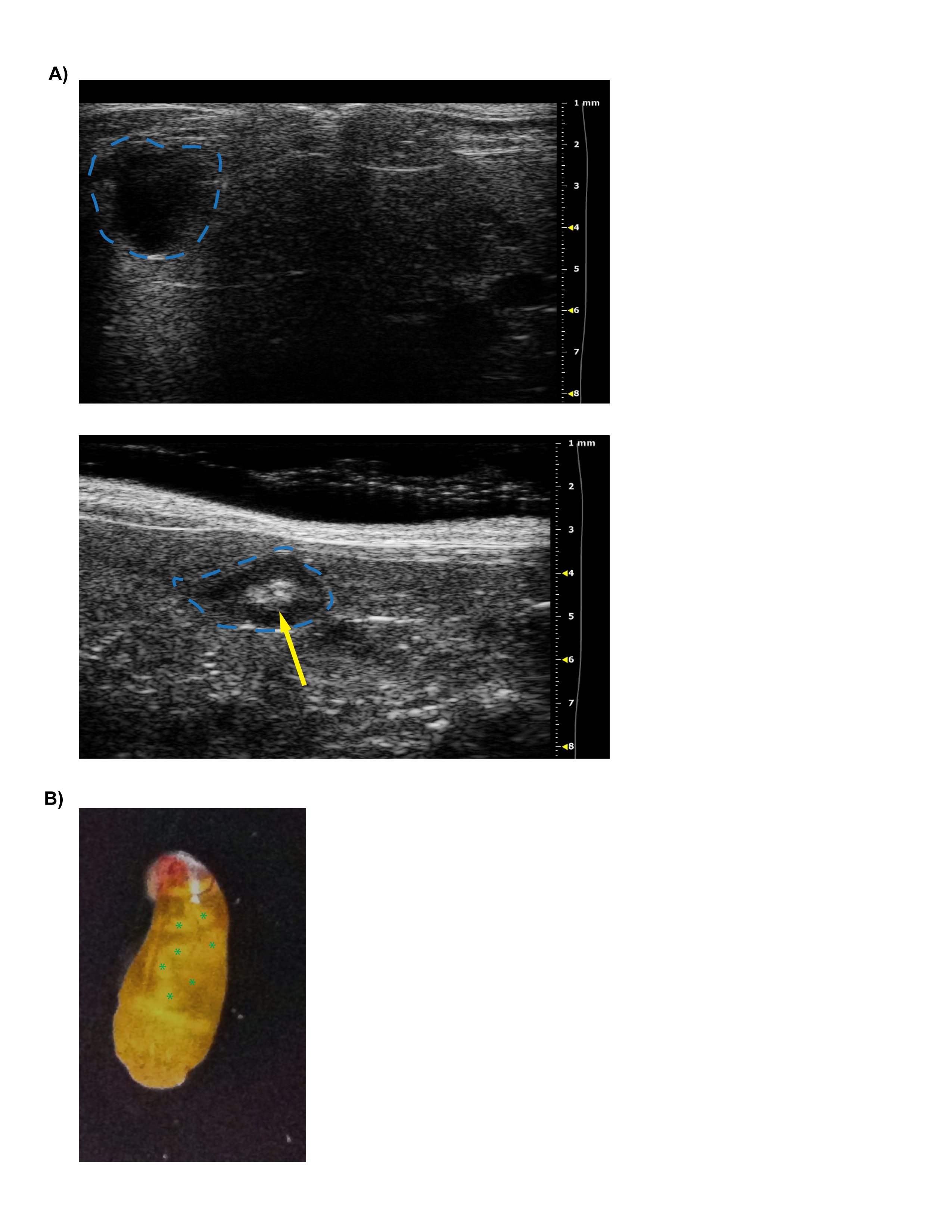

Supplement: Figure S6 — Confirmation of cholesterol gallstone formation. A) Gallbladder ultrasound imaging of mice fed a 10-week control (top) or lithogenic diet (bottom). Blue dashed lines outline radiolucent gallbladders, the yellow arrow indicates a radiopaque gallstone. B) Lithogenic diet-induced gallstone formation (green asterisks) in the gallbladder of a 129X1/SvJ mouse. (TIF) [file ppat.1004527.s006.tif]

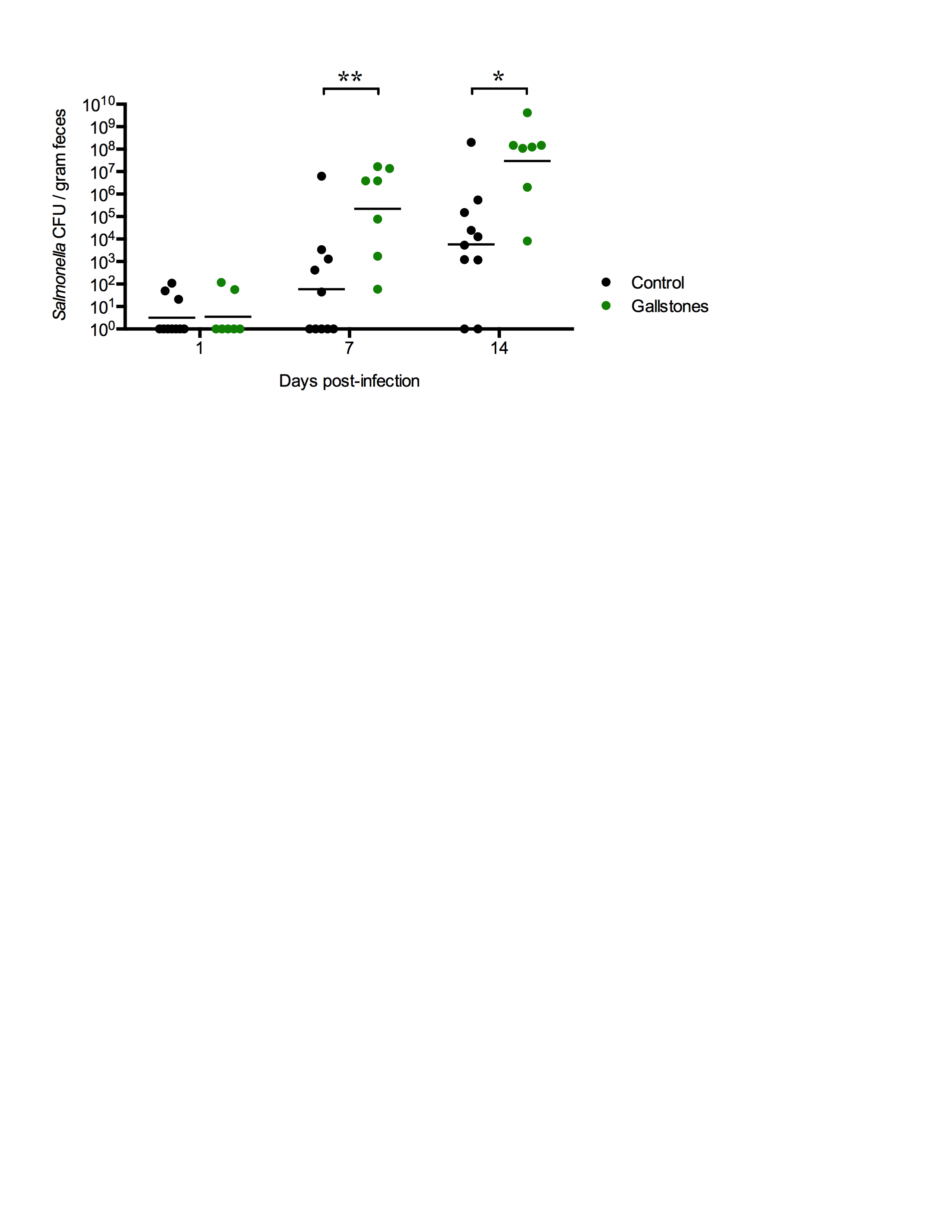

Supplement: Figure S7 — Gallstone formation increases reseeding of the gut by systemic Salmonella . Control mice (black, n = 10) and mice with gallstones (green, n = 7) were infected with 103 SL1344 by IP injection. Fecal Salmonella CFU were enumerated after 1, 7, and 14 days of infection, lines indicate geometric means. Data are representative of two independent experiments. (TIF) [file ppat.1004527.s007.tif]

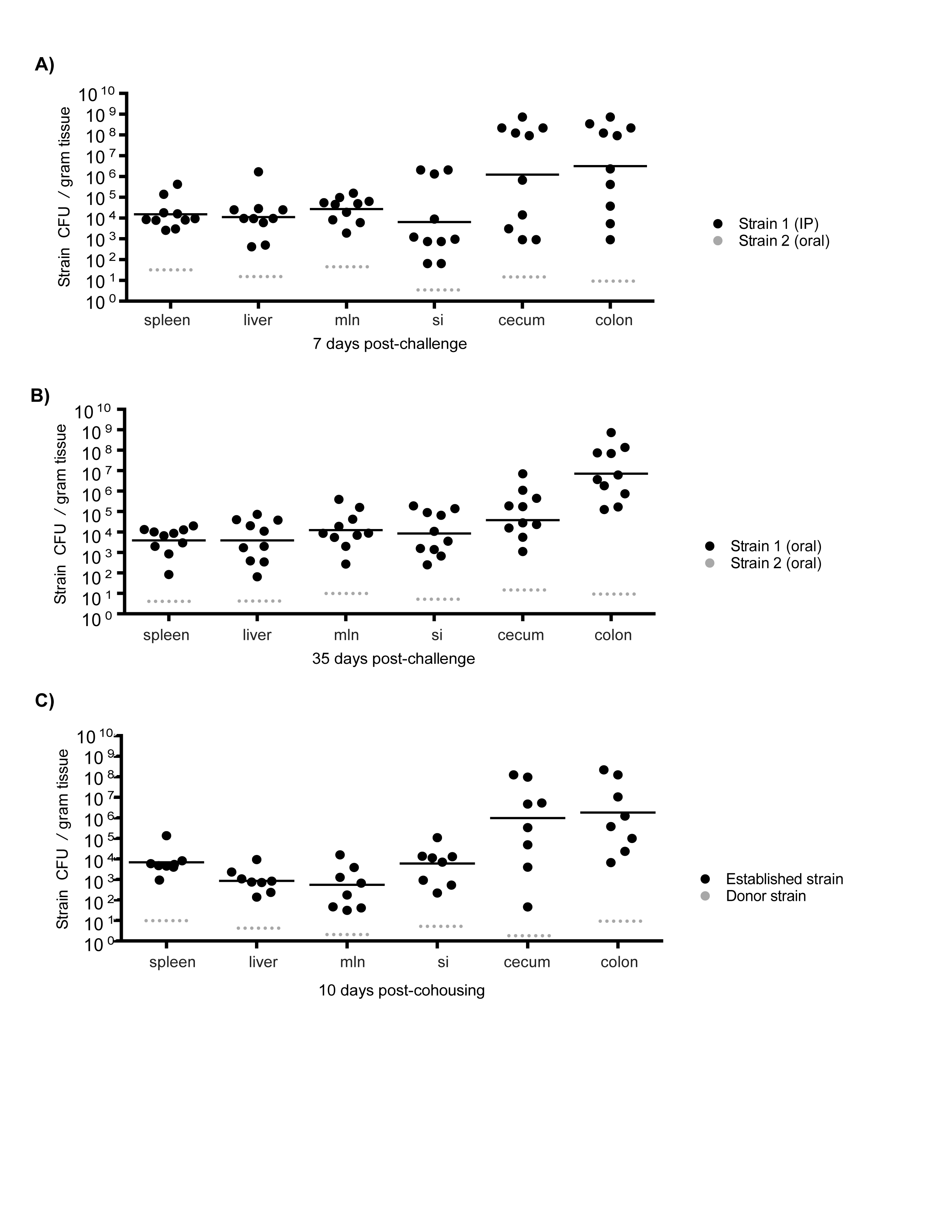

Supplement: Figure S8 — In the presence of an established intestinal strain, challenging Salmonella are cleared from systemic and intestinal tissues. Mice from sequential infections performed in Figure 6. SL1344 was used as the initial strain and SL1344-kanR was used as the challenging strain. Animals were sacrificed at the indicated time points and strain CFU were enumerated in tissues. Challenging strains (gray) were not detected. Limit of detection was determined by both differential plating and patch plating 100 CFU onto antibiotics; indicated by gray dashed lines. A) Mice were infected IP with 103 SL1344 (Strain 1, black) for 35 days, followed by oral challenge with 108 SL1344-kanR (Strain 2, gray). Animals were sacrificed 7 days post-challenge (42 dpi), n = 10. B) Mice were orally infected with 108 SL1344 (Strain 1, black) and challenged orally with 108 SL1344-kanR (Strain 2, gray) after 102 days. Animals were sacrificed 35 days post-challenge (137 dpi), n = 10. C) Recipient mice were orally infected with 108 SL1344 for 14 days before co-housing with a supershedder SL1344-kanR donor. Animals were sacrificed 10 days post-cohousing. Established (SL1344, black) and donor (SL1344-kanR, gray) strain CFU were enumerated, n = 8. (TIF) [file ppat.1004527.s008.tif]

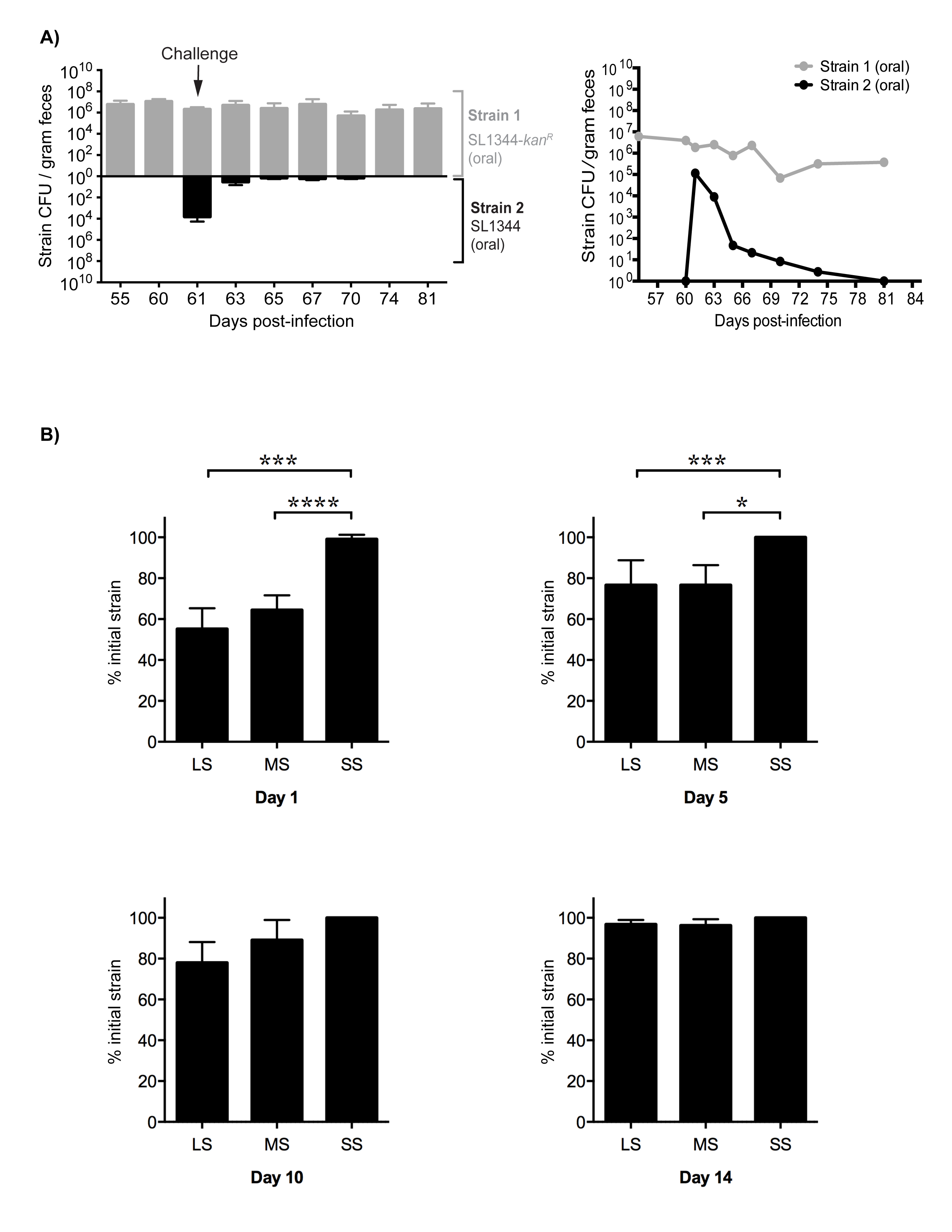

Supplement: Figure S9 — Established strains are resistant to super-colonization, and clearance of the challenging strain occurs more rapidly in super shedders. A) Reciprocal order of strains from those used in sequential oral infections in Figure 6B. Mice were first inoculated with 108 SL1344-kanR (gray) by drinking, which established a persistent infection for 60 days. Animals were subsequently challenged with 108 SL1344 (black) by drinking. Left: Fecal CFU (mean, SD) of established strain SL1344-kanR and challenge strain SL1344 in feces. Right: Geometric mean of strain CFU in feces. Data are representative of two separate experiments (n = 6). B) Analysis of mice orally infected by sequential initial and challenge strains (described in above and in Figure 6B) based on prior shedding status. Percent abundance of the initial strain shed in feces in low (LS, n = 12), moderate (MS, n = 26), and super (SS, n = 8) shedder mice was determined for the specified days post-challenge. *p<0.05, ***p<0.001, ****p<0.0001, unpaired Mann-Whitney tests. (TIF) [file ppat.1004527.s009.tif]

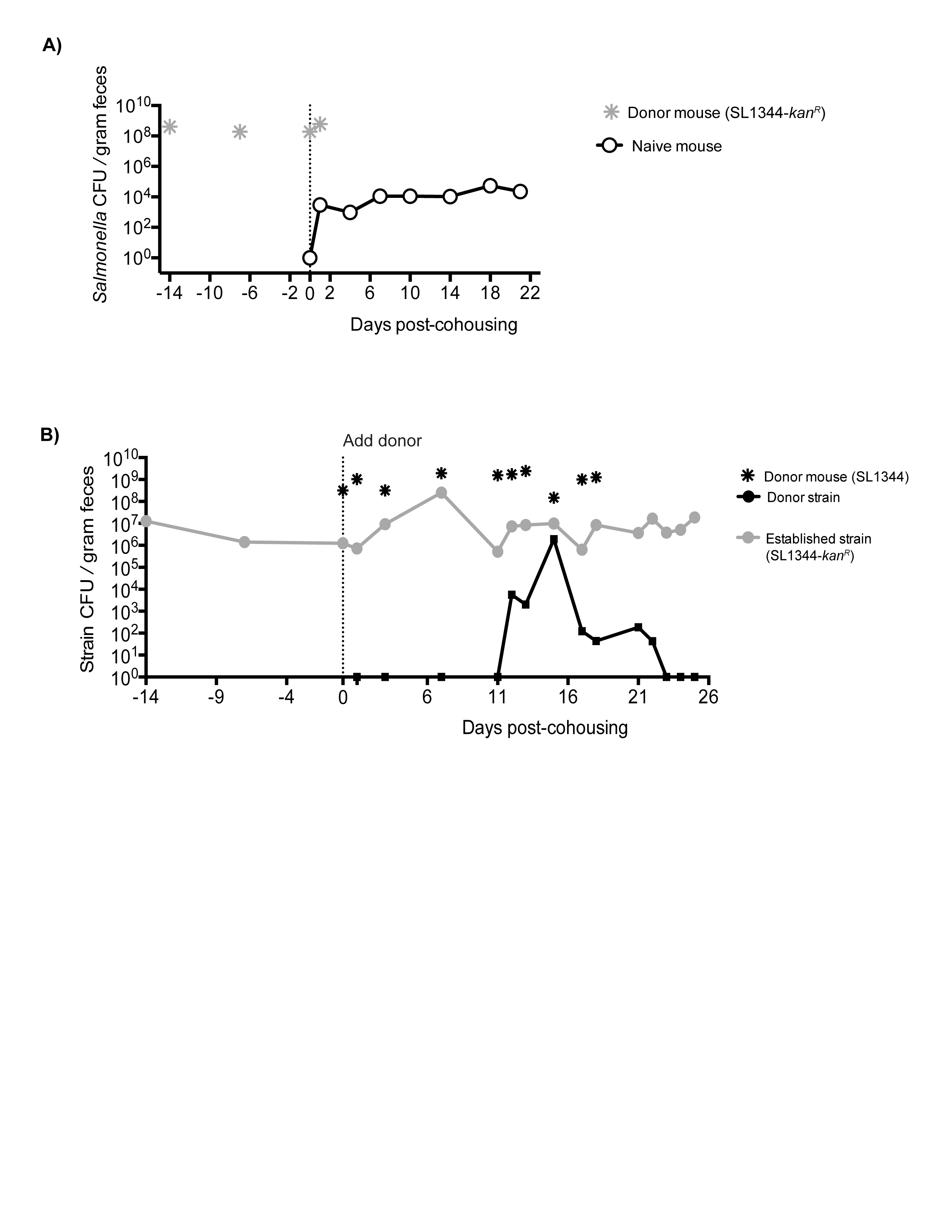

Supplement: Figure S10 — Salmonella SL1344- kanR can be rapidly transmitted to naïve mice, and establishes a persistent intestinal infection exerting intraspecies colonization resistance against SL1344 from an infected donor. A) Super shedder donors rapidly transmit SL1344-kanR to naïve uninfected mice. 14 days prior to cohousing, potential donor mice were infected orally with 108 SL1344-kanR. Fecal Salmonella CFU/gram were tracked and a super shedder donor (gray asterisk) was identified, then cohoused with a recipient naïve mouse for 24 hours. Fecal shedding levels of SL1344-kanR from the recipient mice (open circles) were then tracked over 21 days. Data are representative of two independent experiments (n = 2 donors, 2 naïve recipients). B) Reciprocal order of strains from those used in cohousing experiments in Figure 6C. Mice were first infected orally with 108 of either SL1344 or SL1344-kanR 14 days prior to cohousing. A SL1344 super shedder donor (black asterisk) was cohoused with SL1344-kanR infected recipient mice and removed after 18 days. Geometric means of Salmonella CFU/gram feces in recipient mice shedding the established SL1344-kanR strain (gray) or challenging donor SL1344 strain (black). Data are representative of two independent experiments (n = 2 donors, 6 recipients). (TIF) [file ppat.1004527.s010.tif]

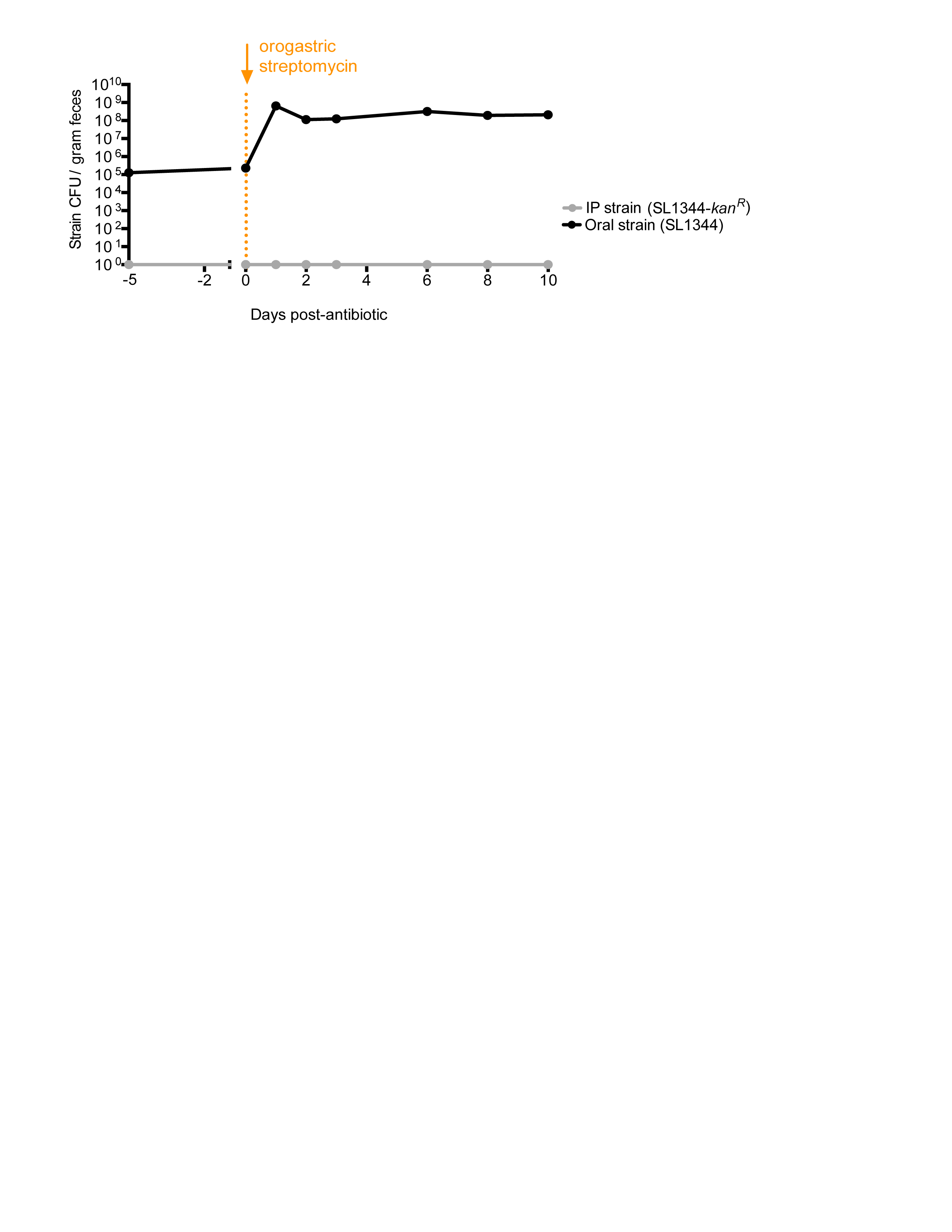

Supplement: Figure S11 — Disruption of microbiota-mediated colonization resistance with streptomycin increases fecal shedding of intestinal Salmonella , but does not permit reseeding by the systemic strain. Mice were co-infected with 108 SL1344 orally and 103 SL1344-kanR IP. A single dose of 5 mg streptomycin in 100 µl water was delivered by oral gavage after 30 days of co-infection (n = 5). Geometric means of oral (black) and IP (gray) strain CFU shed per gram feces. Limit of detection is 10 CFU/gram feces. (TIF) [file ppat.1004527.s011.tif]

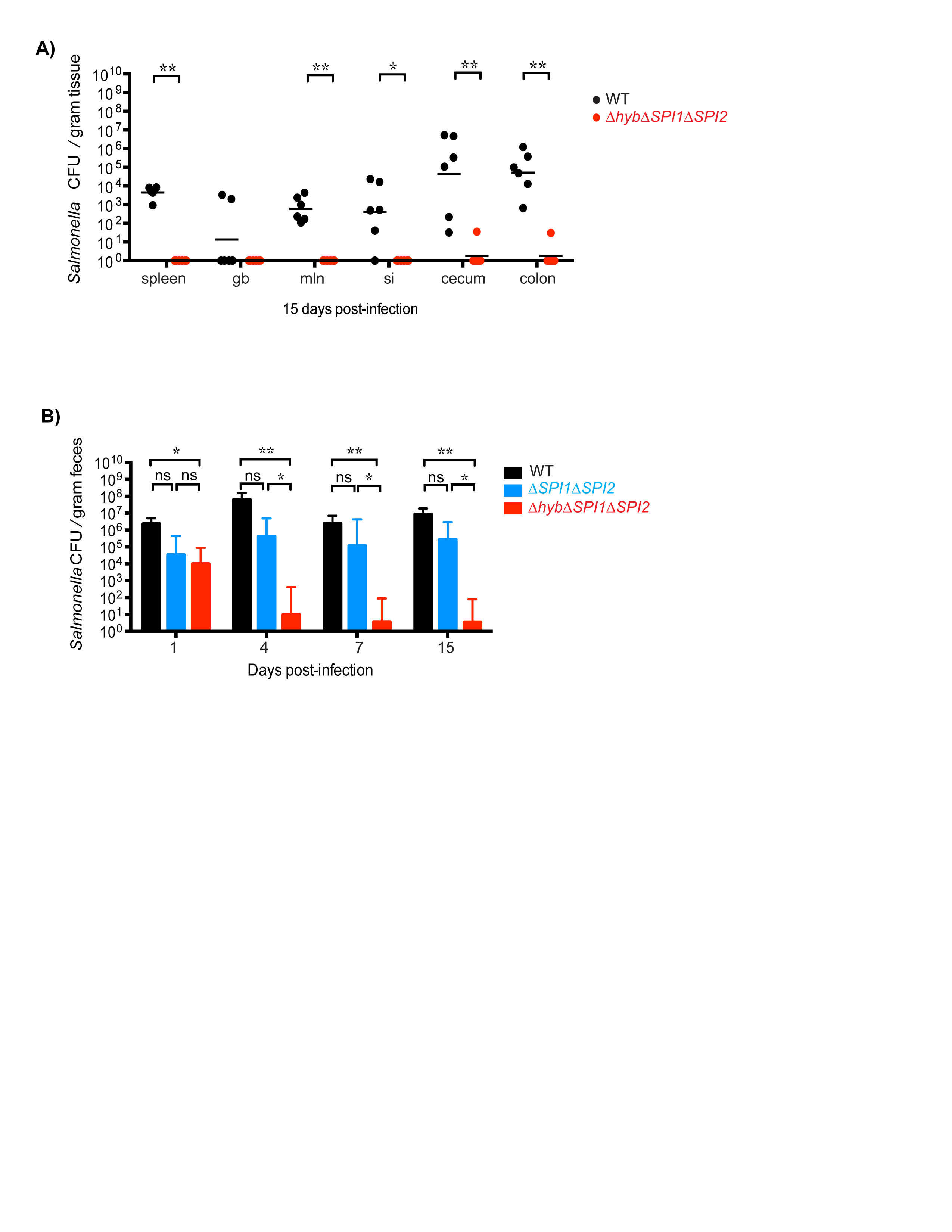

Supplement: Figure S12 — A Salmonella hydrogenase mutant is cleared from feces and tissues after 15 days of infection. Single oral infections were carried out in mice with 108 WT SL1344-kanR (black), ΔSPI1ΔSPI2 (blue), or ΔhybΔSPI1ΔSPI2 (red). Data are representative of two independent experiments (n = 6/group). A) Salmonella CFU in mouse tissues after single oral infections with either WT or ΔhybΔSPI1ΔSPI2 (red) after 15 days of infection. Each circle represents an individual mouse, lines at means. *p = 0.0152, **p = 0.002, unpaired Mann-Whitney tests. B) Fecal shedding of Salmonella was monitored at the specified time points post-infection (mean, SD). No significant differences in Salmonella CFU/gram feces were observed between WT and ΔSPI1ΔSP-2 oral infections. *p<0.0411, **p<0.0022, unpaired Mann-Whitney tests. (TIF) [file ppat.1004527.s012.tif]

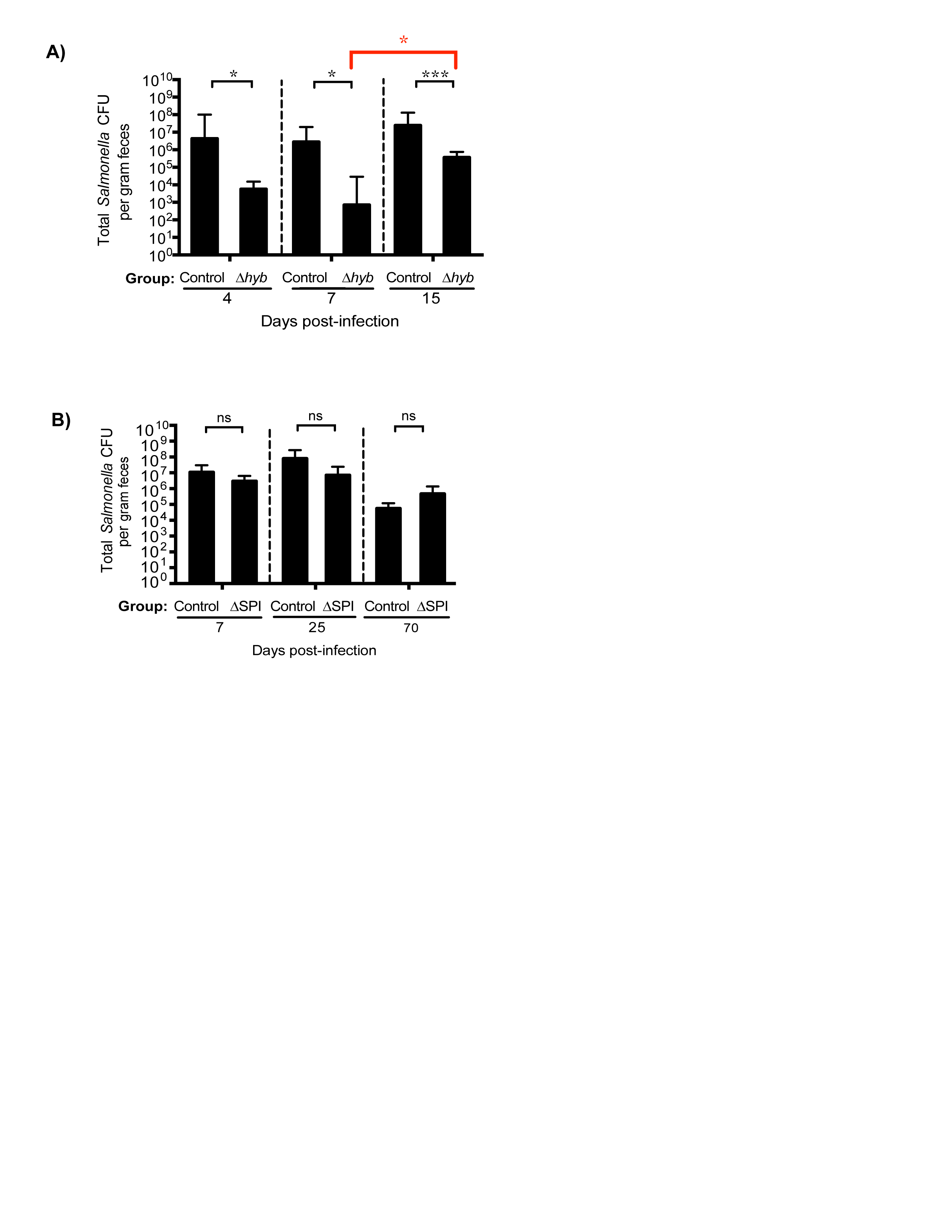

Supplement: Figure S13 — Total Salmonella in feces of mice co-infected with mutant strains. A) Mice in the Δhyb group received 108 ΔhybΔSPI1ΔSPI2 orally and 103 WT SL1344 by IP. Control mice received 108 WT SL1344-kanR orally and 103 WT SL1344 by IP. Data are representative of two independent experiments (control n = 10, Δhyb n = 7). Total Salmonella CFU per gram feces, comprised of both oral and IP strains, detected over 15 days of co-infection for both control and Δhyb mouse groups (mean, SD). Comparison of total fecal Salmonella CFU between day 7 and day 15 in the Δhyb co-infected group is displayed in red (*p = 0.0373). *pday4 = 0.00, *pday7 = 0.0247, p*** = 0.0004, unpaired Mann-Whitney tests. B) Mice in the ΔSPI group received 108 ΔSPI1ΔSPI2 orally and 103 WT SL1344 by IP. Control mice received 108 WT SL1344-kanR orally and 103 WT SL1344 by IP. Data are representative of two independent experiments (n = 10/group). Total Salmonella CFU per gram feces, comprised of both oral and IP strains, detected at the specified time points throughout 70 days of co-infection for both control and ΔSPI mouse groups (mean, SD). ns = not significant, unpaired Mann-Whitney tests. (TIF) [file ppat.1004527.s013.tif]
